# Stochastic processes dominate community assembly of ectomycorrhizal fungi associated with *Picea crassifolia* in the Helan Mountains, China

**DOI:** 10.3389/fmicb.2022.1061819

**Published:** 2023-01-12

**Authors:** Xuan Zhang, Yonglong Wang, Ying Xu, Busayo Joshua Babalola, Simin Xiang, Jianjun Ma, Yun Su, Yongjun Fan

**Affiliations:** ^1^Faculty of Biological Science and Technology, Baotou Teacher's College, Baotou, China; ^2^State Key Laboratory of Mycology, Institute of Microbiology, Chinese Academy of Sciences, Beijing, China; ^3^College of Life Sciences, Langfang Normal University, Langfang, Hebei, China; ^4^Helan Mountains National Nature Reserve Administration of Inner Mongolia, Alxa League, China; ^5^School of Life Science and Technology, Inner Mongolia University of Science and Technology, Baotou, China

**Keywords:** ectomycorrhizal fungi, *Picea crassifolia*, community assembly, stochastic processes, dispersal limitation

## Abstract

**Introduction:**

Understanding the underlying mechanisms of microbial community assembly is a fundamental topic in microbial ecology. As an integral part of soil organisms, ectomycorrhizal (EM) fungi play vital roles in ecosystems. *Picea crassifolia* is an important pine species in the Helan Mountains in Inner Mongolia, China, with high ecological and economic values. However, studies of EM fungal diversity and mechanisms underlying community assembly on this pine species are limited.

**Methods:**

In this study, we investigated EM fungal communities associated with *P. crassifolia* from 45 root samples across three sites in the Helan Mountains using Illumina Miseq sequencing of the fungal rDNA ITS2 region.

**Results:**

A total of 166 EM fungal OTUs belonging to 24 lineages were identified, of which *Sebacina* and *Tomentella-Thelephora* were the most dominant lineages. Ordination analysis revealed that EM fungal communities were significantly different among the three sites. Site/fungus preference analysis showed that some abundant EM fungal OTUs preferred specific sites. Ecological process analysis implied that dispersal limitation and ecological drift in stochastic processes dominantly determined the community assembly of EM fungi.

**Discussion:**

Our study indicates that *P. crassifolia* harbors a high EM fungal diversity and highlights the important role of the stochastic process in driving community assembly of mutualistic fungi associated with a single plant species in a semi-arid forest in northwest China.

## Introduction

Ecological research that unravels the ecological processes of microorganisms is a key topic but is poorly understood (Dini-Andreote et al., 2015; Zhou and Ning, 2017). Based on the niche-based theory and neutral theory, the deterministic and stochastic processes are generally used to describe the ecological processes of the community assembly (Dini-Andreote et al., 2015; Zhou and Ning, 2017). Accumulating evidence also suggests that the two theories are not independent. More specifically, the deterministic and stochastic processes act concurrently to regulate the community assembly (Sloan et al., 2006; Bahram et al., 2016). As a common approach based on Stegen et al. (2012), five ecological processes were included in the deterministic and stochastic processes in driving community assembly, that is, the deterministic process includes the heterogeneous selection and homogeneous selection, while the stochastic process harbors dispersal limitation, homogenizing dispersal, and drift. In detail, heterogeneous selection is defined as the selection derived from different abiotic and biotic variables in the community and results in more dissimilarity among communities, by contrast, homogeneous selection implies the selection under similar abiotic and biotic conditions leading to more similar communities. Dispersal limitation claims that the movement and colonization of individuals in a new habitat are restricted; homogenizing dispersal indicates a high rate of dispersal and effective colonization of individuals among communities, which leads to more similar communities; and drift means that the relative abundance of species randomly change in the community over the time due to stochastic process of inherence, such as speciation, random birth, and death. Recently, the relative roles of deterministic and stochastic processes on the microbial community, as well as the contributions of the five ecological processes in controlling the community assembly of microbes, have been extensively investigated. Using a neutral community model (NCM) and a normalized ratio index (NST), for example, Chen et al. (2021) found that the assembly of the microbial community in termite mounds was strongly driven by the deterministic process. Chen et al. (2019) demonstrated that stochastic processes were sufficient in driving river microeukaryotic communities. In some studies, based on the null model analysis proposed by Stegen et al. (2012), detailed ecological processes driving microbial community assembly have been investigated. For example, Zhang et al. (2021) indicated a determinant role of dispersal limitation in the stochastic process on fungal community assembly in mangrove sediments in southern China. Jiao et al. (2021) found that in the agricultural fields, the fungal community of abundant groups was mainly governed by dispersal limitation in the stochastic process, while the homogeneous selection in the deterministic process was the dominant ecological process in controlling community of the rare fungi. Additionally, for the marine benthic archaeal communities, dispersal limitation in the stochastic process was the main community ecological process (Liu et al., 2020). In the study of the assembly mechanism of bacterial communities on plastisphere in water, it was found that the bacterial communities of plastic balls were mainly driven by the stochastic process of drift and dispersal limitation (Sun et al., 2021). These studies have further clarified the different ecological processes and their relative importance in controlling some microbial communities. However, it is obvious that the microbes in the studies mentioned above focused on either bacteria, archaea, and/or fungi in the water and soil environments, that is, “Free-living” microbes. Thus, an important question remains about ecological processes that underlie the assembly of symbiotic microbes such as mycorrhizal fungi, and it remains uncertain and deserves further exploration since they play a vital role in ecosystems. Mycorrhizae is a typical symbiosis formed between soil fungi of certain groups and host plants. There is increasing evidence that mycorrhizal fungi play important roles in host growth, development, and resistance to stress and can provide host plants with the required nitrogen and phosphorus to increase nutrient absorption (Smith and Read, 2012; van der Heijden et al., 2015). Ectomycorrhizal (EM) fungi are certain groups in mycorrhizal fungi, which could form symbiosis (i.e., ectomycorrhizae) with 30 lineages of angiosperms and gymnosperms, accounting for 2% of terrestrial plants and mostly trees. The ratio of EM host plants is low, but the plants (e.g., Pinaceae, Fagaceae, Betulaceae, and Salicaceae) played important roles in the ecosystems, particularly in the forests (Tedersoo et al., 2020).

A growing number of studies have focused on the relative effects of environmental filtering (e.g., host plant phylogeny, soil factors, and climate factors) and dispersal limitations (e.g., spatial distance) on EM fungal community (Gao et al., 2013, 2015; Tedersoo and Smith, 2013; Wu et al., 2018; Wang et al., 2019, 2021a,b). For example, several studies indicated that EM fungal community was mainly affected by host phylogeny and by spatial distance, soil factors, and climate combination factors (Ishida et al., 2007; Tedersoo et al., 2010; Wang et al., 2019, 2021a,b). These studies mainly reveal the effect of abiotic and abiotic variables on the EM fungal community, but the community assembly from the aspect of ecological processes and their relative importance remains unclear and needs further study.

The Helan Mountains are located in the transitional zone between grassland and desert types of vegetation (Jiang et al., 2007). The research site in the Helan Mountains Range is a unique geographic location that connects the flora and climate of the Inner Mongolian Plateau in Northern China. *Picea crassifolia* Kom. forests account for 90% of the total forest cover of the Helen Mountains Range, but the EM fungal diversity and ecological processes underlying the community assembly of EM fungi on this plant species have received limited attention. With this in mind, we attempted to provide the first insight into *P. crassifolia* EM fungal diversity in this nutrient-and water-limited boreal area. We addressed the following questions: (1) How about EM fungal diversity and community composition of *P. crassifolia* in the Helan Mountains? and (2) What ecological processes determine the community assembly of EM fungi associated with *P. crassifolia* in the Helan Mountains?

## Materials and methods

### Sampling and sequencing

Our study was carried out in the Helan Mountains National Nature Reserve of Inner Mongolia, China. The nature reserve is located in a temperate continental semi-arid climate zone, with a mean annual temperature of 3.01°C and a mean annual precipitation of 243 mm based on the climate data from the WorldClim dataset (Hijmans et al., 2005). The nature reserve was protected well and without any anthropogenic disturbances. To obtain a comprehensive map of EM fungal diversity in the Helan Mountain, three sites typical for *P. crassifolia* (BeiSi: HLSBS, HaLawugo: HLWG, and NanSi: HLSNS), which were separated by about 20 km from each other were selected for our study. In these three sites, *P. crassifolia* was the most dominant woody plant, and about 60 years old, with very less shrubs under the pine forests. The sample collection was approved by the Helan Mountains National Nature Reserve of Inner Mongolia, China. Sampling work was conducted in the summer of 2017 (growing season). At each site, 13–16 plant individuals of *P. crassifolia* were selected. Among them, the plant was carefully identified by Professor Yongjun Fan from Inner Mongolia University of Science and Technology based on the descriptions in Flora of China, a voucher specimen was deposited in the Herbarium of Faculty of Biological Science and Technology, Baotou Teacher’s College, with an accession number BTSY-PC0150. The individuals were away from each other by at least 10 m to ensure independence (Lilleskov et al., 2004). The fine roots including mycorrhizal and non-mycorrhizal were excavated by tracing the roots to the base of the trunk of each plant at three points and merged as one sample (the distance from the trunk was within about 50 cm). The root samples were transferred to the laboratory in an ice box within 24 h and stored at −20°C until processing. Additionally, the tree age, diameter at breast height, and altitude were similar in the three sites to avoid their influence on fungal communities. In total, 45 root samples were collected in this study. The latitude, longitude, and altitude for each site were recorded by a portable global positioning system (GPS Jisibao G330, Bejing, China). Details on geographical location and climatic conditions can be found in Supplementary Table 1.

In the laboratory, the fine roots were first washed carefully with sterilized water and then cut into about 2 cm-long fragments. The EM root tips were identified and picked under the stereomicroscope based on their morphological characteristics such as emanating hyphae, color, and shapes. The EM root tips were thicker than non-mycorrhizal roots, and monopodial or branching, with brown, yellow, and black colors, and some EM root tips harbored clear external hyphae. Here, we randomly selected 200 healthy EM root tips from each sample under a stereo-microscope for total genomic DNA extraction. A detailed description of DNA extraction can be found in the method of Gao et al. (2013), and other specific methods such as PCR protocol can be found in Ihrmark et al. (2012) and Wang et al. (2019). Briefly, the ITS2 region of fungal was amplified using a two-step PCR in a Veriti 96-well Thermal Cycler (Applied Biosystems, Foster City, United States). The PCR products were purified using a PCR Purification Kit (NO:28104). All purified PCR products were mixed in equimolar amounts by Qubit 2.0 Fluorometer (Thermo Fisher Scientific, Waltham, MA, United States) and then sequenced by the Illumina MisSeq PE 250 (Illumina, San Diego, CA, United States) in the Environmental Genome Platform of Chengdu Institute of Biology, Chinese Academy of Sciences, China.

### Bioinformatic analysis

Raw data were processed and analyzed by using the Quantitative Insights in Microbial Ecology platform (Knight et al., 2010), and low-quality reads with an average quality score *<* 20, no valid primer sequence or barcode sequence, uncertain bases *>*6, or length *<* 250 bp were removed. The ITS2 region of the filtered sequences was extracted using the ITSx software (Bengtsson-Palme et al., 2013), and redundancy sequences were removed by VSEARCH software (Rognes et al., 2016) before the downstream analysis. Chimeras were identified and removed using USEARCH 11 (Edgar et al., 2011). After that, sequences were clustered into OTUs by using the UPARSE pipeline (Edgar, 2013) with a 97% sequence similarity cutoff. The representative sequence (most abundant sequence) of each OTU was aligned against the UNITE database (v. 8.2, release date: 02.04.2020) by using the basic local alignment search tool (BLAST; Altschul et al., 1990). Fungal OTUs and taxonomic identification were determined according to the criteria proposed by Tedersoo et al. (2014). EM fungal OTUs and lineages were identified based on Tedersoo and Smith (2013) and Tedersoo et al. (2014). To avoid the effect of different sequencing depths on the subsequent analysis, EM fungal data were normalized to 2,621 (the minimum sequence number among 45 samples) by using the “sub.sample” command in Mothur software (Schloss et al., 2009). The representative sequences of each EM fungal OTU were deposited in the European Nucleotide Archive under study accession no. OU989054–OU989219. Information on EM fungi in the present study is shown in Supplementary Table 2.

### Statistical analysis

All statistical analysis was performed in R3.6.3 (R Development Core Team, 2019). The principal analysis process was as follows: (1) Alpha diversity for EM fungal analysis: The alpha diversity indices were calculated for each sample in the vegan package, which included OTU richness, Shannon-Wiener, Simpson, Chao1, and abundance-based coverage estimator (ACE) index. The nonparametric Wilcoxon test was used to compare the difference in alpha diversity between sites. (2) Beta diversity for EM fungal analysis: The EM fungal OTUs count data were first Hellinger transformed, and the Bray-Curtis distance matrix of the EM fungal community was constructed (Clarke et al., 2006). Based on Bray–Curtis dissimilarity matrices, the principal coordinate analysis (PCoA) was used to visualize the differences in EM fungal communities at three sites, and the ordiellipse function was used to fit the 95% CIs of sites onto the PCoA ordination. Subsequently, permutational multivariate ANOVA (PerMANOVA) with 999 permutations was adopted to evaluate the significance of the difference in the EM fungal communities among the three sites. Altitude and climatic variables were fitted into the PCoA ordination to investigate their effect on EM fungal community using the environmental fitting test. (3) Site/EM fungus preferences analysis: The analysis mainly refers to Toju et al. (2016) and Wang et al. (2021b), and a detailed description is available in the Supplementary methods. (4) Community assembly for EM fungal analysis: To determine the potential importance of stochastic processes in the assembly of EM fungal communities, the NCM was used to account for the relationship between OTU detection frequency and relative abundance. Phylogeny and null model analysis have been popularly used to understand the relative importance of deterministic and stochastic processes in community assembly in recent years (Stegen et al., 2012; Wang et al., 2021a). According to the method described in a study by Stegen et al. (2012), a β-mean nearest taxon distance (βMNTD) was used to calculate the phylogenetic turnover between samples and to calculate the standard deviation of the observed βMNTD value from the null distribution of βMNTD headquarters based on the standardized estimate β-nearest taxon index (βNTI) of MNTD in the picante packages (Liu et al., 2020; Wang et al., 2021a). βNTI values >2 or < −2 indicate a deterministic process, and it mainly includes the heterogeneous selection and homogeneous selection. |βNTI| values <2 represent stochastic processes that include homogenizing dispersal, dispersal limitation, and drift (Liu et al., 2020; Wang et al., 2021a). To further investigate the relative contribution of heterogeneous selection, homogeneous selection, homogenizing dispersal, dispersal limitation, and drift in determining EM fungal community assembly, the infer community assembly mechanisms by phylogenetic-bin-based null model analysis (iCAMP), which is a framework that quantitatively infers the mechanism of community assembly proposed by Ning et al. (2020), was adopted in this study. In iCAMP, EM fungal OTUs are divided into different bins by the phylogenetic relationship of OTUs, and the value of beta net relatedness index (βNRI) and Raup–Crick metric (RC) was calculated for each bin based on the null model to quantify various ecological process. Here, βNRI < −1.96 is regarded as the percentage of homogeneous selection, and βNRI > +1.96 is regarded as the percentage of heterogeneous selection. Additionally, |βNRI| ≤ 1.96 is determined by the RC value, RC < −0.95 is considered the percentage of homogenizing dispersal, RC > +0.95 is considered as dispersal limitation, |RC| ≤ 0.95 is considered as drift, diversification, and weak percentage of selection or weak dispersion. To investigate the influence of site-preference OTUs on community assembly, the iCAMP analysis was re-conducted after removing the significant OTUs with site preference. The data and analysis codes used in this study have been provided as Supplementary material (Supplementary Table 3 and Supplementary codes).

## Results

### Ectomycorrhizal fungal diversity

After quality filtering, in total 4,587 non-redundant ITS2 sequences were obtained from 897,581 raw sequences. Then, in total 4,193 non-chimeric sequences were obtained from non-redundant sequences. These sequences were clustered into 713 (857,370) OTUs, of which 166 (555,707) OTUs were identified as EM fungi. After the rarefication, a normalized dataset containing 166 OTUs was still retained for subsequent analysis (Supplementary Figure 1). Among the 166 OTUs, 29 belonged to Ascomycota (17.470% of total EM fungal reads) and 137 belonged to Basidiomycota (82.530%).

**Figure 1:**
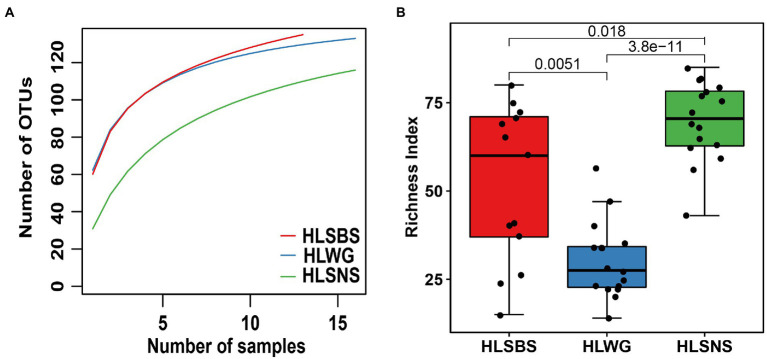
Alpha diversity index of the ectomycorrhizal fungal community of *Picea crassifolia*. Rarefaction of ectomycorrhizal fungal operational taxonomic units (OTUs) in three sites **(A)**; Richness index **(B)**. Data were analyzed using nonparametric method followed by the Wilcoxon test for **(B)**. HLSBS, HeLanShan-BeiSi; HLWG, HaLaWuGo; and HLSNS, HeLanShan-NanSi.

The rarefaction curves for the observed EM fungal OTUs in root samples of three sites did not reach an asymptote, indicating that the annotation results cannot fully reflect the true situation of the EM fungal community in the sample, and more sample collection could result in more undiscovered OTUs (Figure 1A). A total of 166 EM fungal OTUs were found from 45 samples in the present study, and the richness index and the Shannon index were 50.511 ± 3.334 (mean ± SE) and 3.108 ± 0.040 (mean ± SE), respectively. We analyzed the alpha diversity of the EM fungal community of *P. crassifolia* in three sites. The richness of EM fungi of HLSBS and HLSNS was significantly higher than that of HLWG (Wilcoxon: *p* < 0.05, Figure 1B). Similarly, significant differences were also observed between sites in terms of diversity index including Shannon-Wiener, Simpson, Chao1, and ACE (Wilcoxon: p < 0.05, Supplementary Figure 2). The above results indicated that there were significant differences in the diversity of EM fungi in the three locations. The upset diagram showed that the number of OTUs was different in the three sites, namely, HLSNS, HLWG, and HLSBS, and the values were 126, 107, and 135 respectively, of which 68 OTUs were shared by the three sites, accounting for 41% of total OTUs numbers. Furthermore, each of the sites harbors unique OTUs, and 16, 11, and five fungal OTUs only existed on HLSNS, HLWG, and HLSBS, respectively, accounting for 19.28% of total OTUs number, and 66 OTUs (39.76%) were shared by two sites (Supplementary Figure 3).

### Ectomycorrhizal fungal community

**Figure 2:**
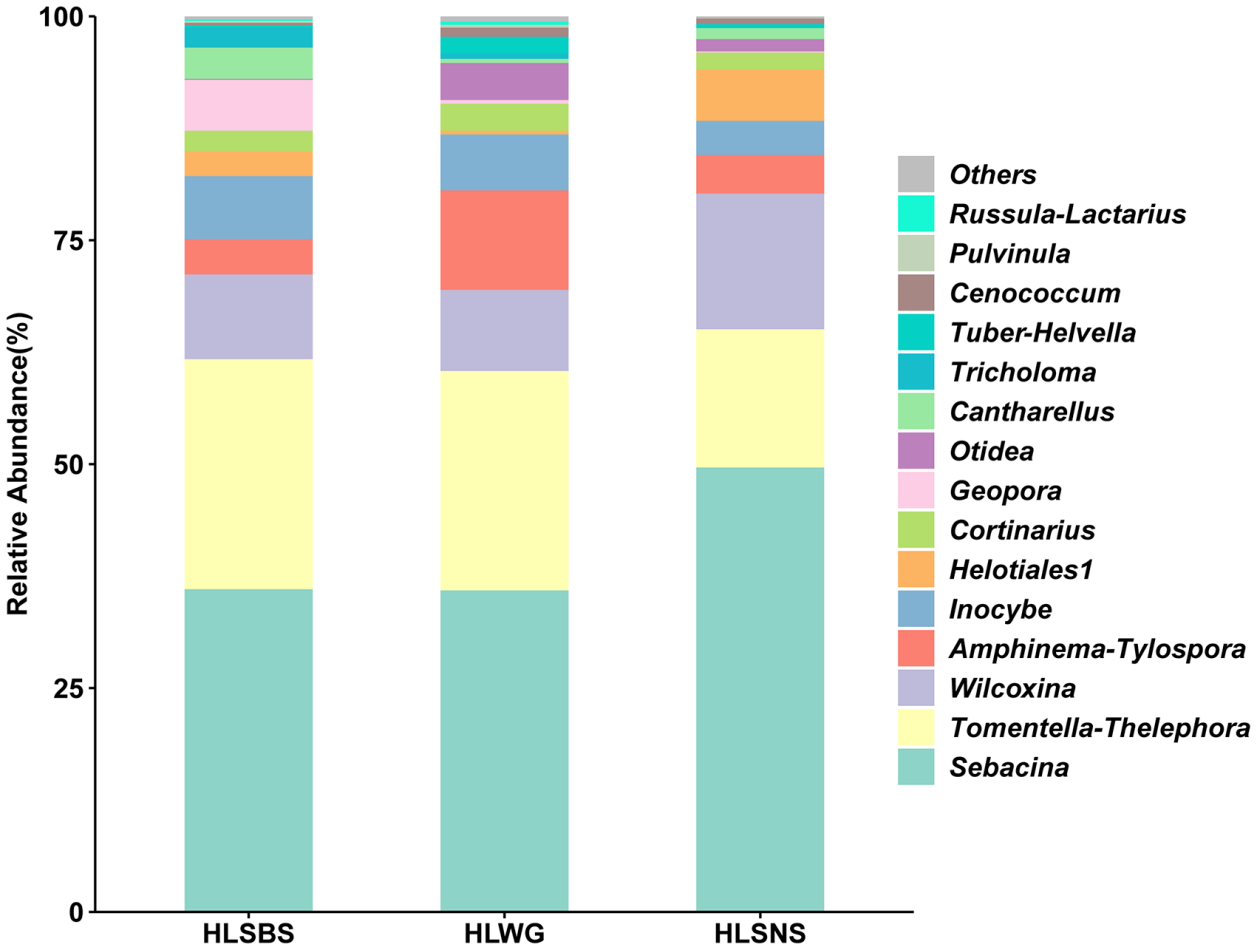
Ectomycorrhizal fungal lineage and their relative abundance in three sites. Here, we only showed the top 15 EM fungal lineages. HLSBS, HeLanShan-BeiSi; HLWG, HaLaWuGo; and HLSNS, HeLanShan-NanSi.

In this study, a total of 24 EM fungal lineages were found in the root samples of *P. crassifolia*. The relative abundances of the top 15 EM fungal lineages in the three sites are shown in Figure 2. We found that *Sebacina*, *Tomentella-Thelephora*, *Wilcoxina*, and *Amphinema-Tylospora* were the most abundant lineages of EM fungi, which is consistent with the study of Wang et al. (2021a) to a certain extent. Subsequently, based on the relative abundance of EM fungal lineages, we compared the species composition of the three sites HLSBS, HLWG, and HLSNS. The relative abundances of *Sebacina* and *Wilcoxina* were relatively higher in HLSNS than in HLSBS and HLWG, and the relative abundance of *Sebacina* in HLSNS was significantly higher than in HLWG (Wilcoxon: *p* < 0.05, Supplementary Figure 4); by contrast, the relative abundance *Tomentella-Thelephora* in HLSNS was relatively lower than in HLSBS and HLWG, while significant difference was not observed between sites (Wilcoxon: *p* > 0.05, Supplementary Figure 4).

**Figure 3:**
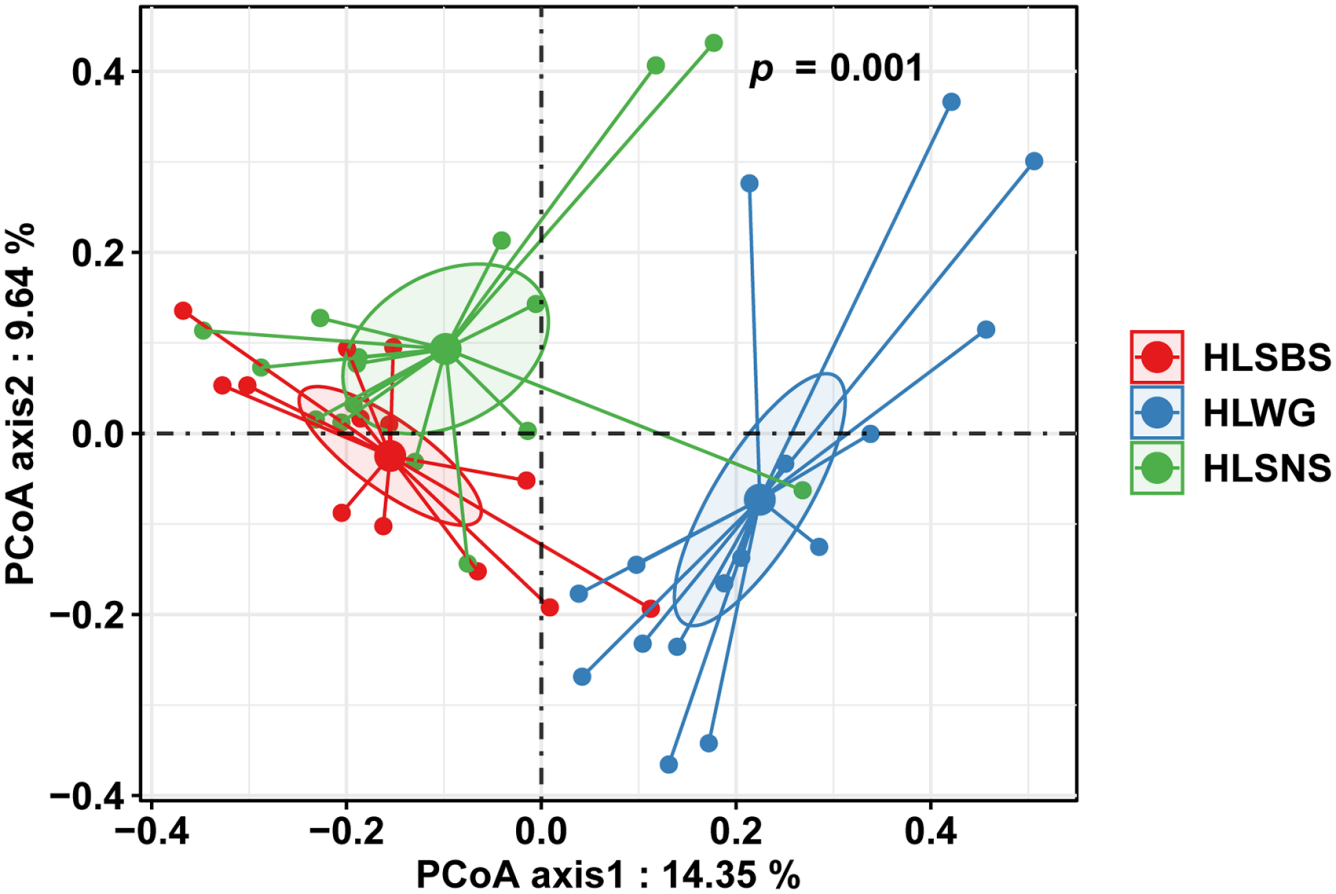
Principal coordinate analysis (PCoA) of ectomycorrhizal (EM) fungal community composition based on Bray–Curtis similarity. EM fungi were clustered, and the center of gravity was computed for each site. HLSBS, HeLanShan-BeiSi; HLWG, HaLaWuGo; and HLSNS, HeLanShan-NanSi.

**Figure 4:**
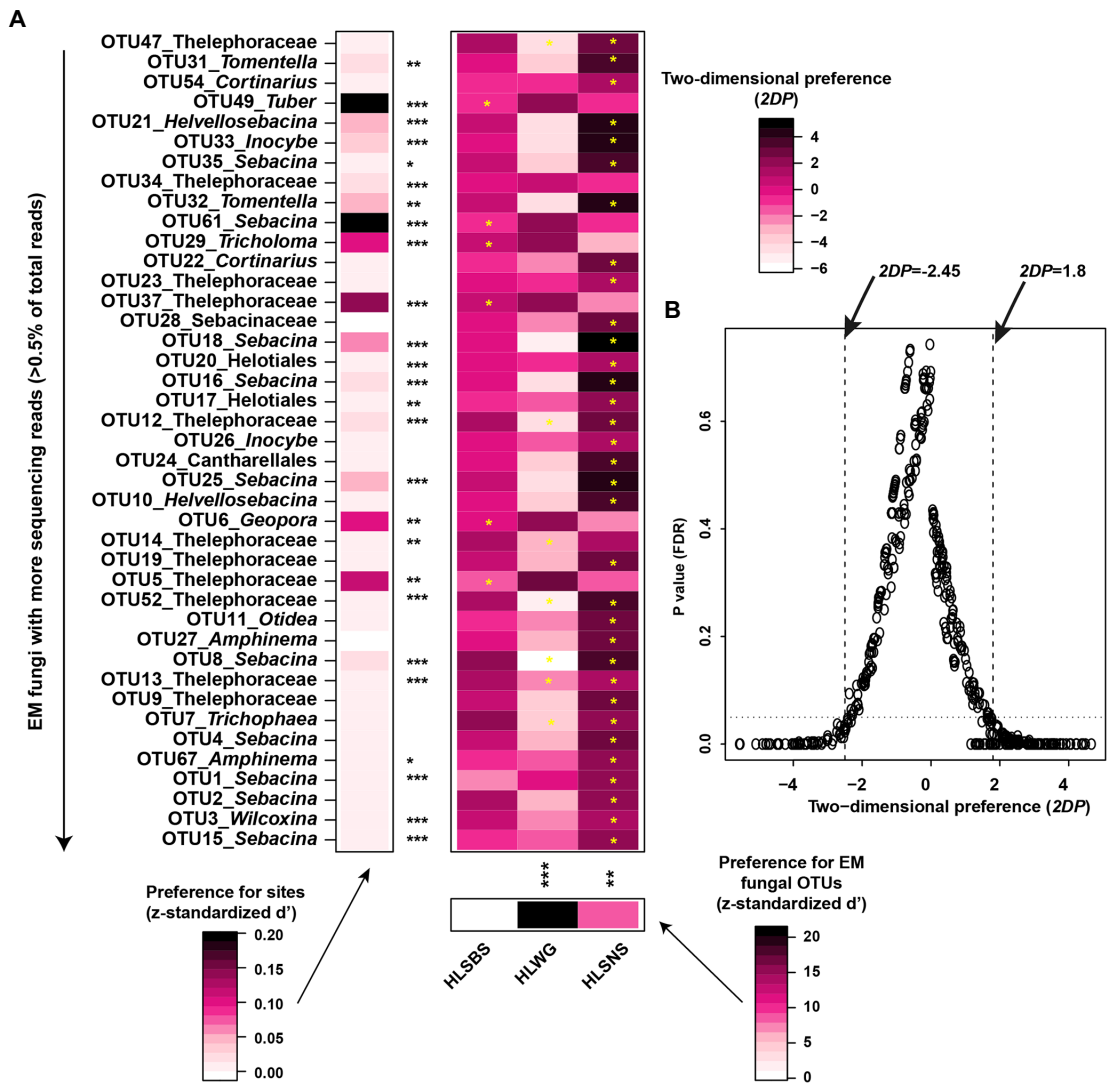
Preferences of site-fungus associations. **(A)** Standardized d’ estimates of preferences for ectomycorrhizal (EM) fungal operational taxonomic units (OTUs) for sites (columns). Similarly, the standardized d’ estimate of preferences for sites is indicated for each of the observed fungal OTUs (row). Each cell in the matrix indicates a two-dimensional preference (2DP) estimate and evaluates to what extent each pair of each site–fungus association was observed (counts) more or less frequently than would be expected by chance. **(B)** Relationship between 2DP and FDR adjusted *p* values, 2DP values larger than 1.80 represented strong preferences. The *p* value obtained from the preference analysis was generated by the one-tail method and adjusted based on the false discovery rate (FDR). Significance: ^*^p < 0.05, ^**^p < 0.01, and ^***^p < 0.001. HLSBS, HeLanShan-BeiSi; HLWG, HaLaWuGo; and HLSNS, HeLanShan-NanSi.

The principal coordinates analysis (PCoA) at the OTU level indicated that the EM fungal communities were significantly different among three sampling sites (PerMANOVA, pseudo-*F* = 2.93, *R^2^* = 0.122, *p* = 0.001), particularly the fungal community in HLWG clearly segregated from those in the other two sites (Figure 3). The environmental fitting test indicated that the mean annual precipitation (MAP) significantly affects EM fungal community composition (Supplementary Figure 5). The preference analysis on site/fungus association indicated that 26 of 41 (63.41%) abundant EM fungal OTUs (> 0.5% of total reads) significantly occurred in specific sites; however, the fungal OTUs showed different preferences for each site (Figure 4). For instance, OTU5_Thelephoraceae, OTU6_*Geopora*, OTU37_Thelephoraceae, OTU61_*Sebacina*, OTU29_*Tricholoma*, and OTU49_*Tuber* showed a stronger preference for HLSBS; OTU25_*Sebacina,* OTU20_Helotiales, OTU18_*Sebacina*, OTU32_*Tomentella,* OTU35_*Sebacina,* OTU33_*Inocybe,* and OTU31_*Tomentella* showed a stronger preference for HLSNS; OTU14_Thelephoraceae, OTU12_Thelephoraceae, OTU52_Thelephoraceae, OTU8_*Sebacina,* and OTU7_*Trichophaea* showed a stronger preference for HLWG (Figure 4). In summary, HLSNS, HLWG, and HLSBS harbored 33 (26.829%), 7 (5.691%), and 6 (4.878%) of 123 pairs of site and fungus that exhibited a remarkably strong preference, respectively (Figure 4).

### Ectomycorrhizal fungal community assembly processes

**Figure 5:**
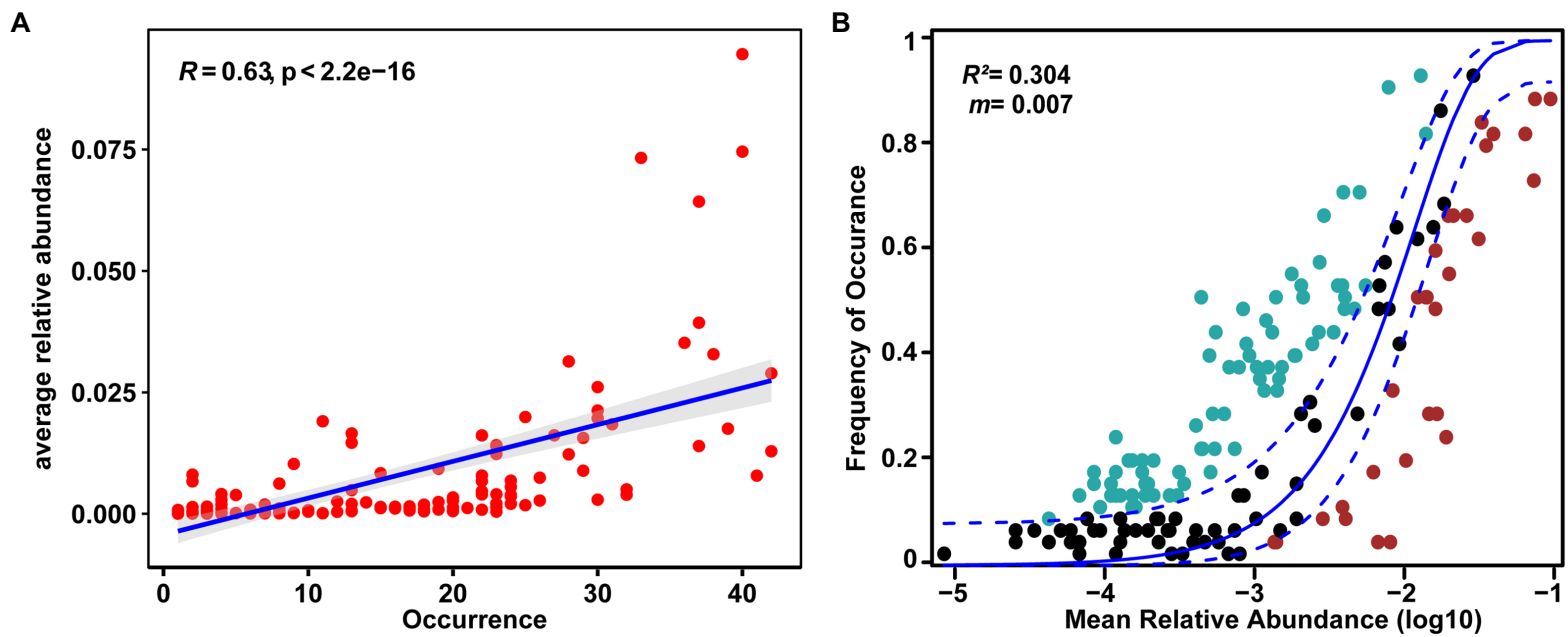
Relationships between occurrence and abundance of ectomycorrhizal (EM) fungal OTUs. **(A)** Abundance–occupancy relationship based on all OTUs. Spearman’s rank correlation was calculated between average relative abundance and the number of sites occurred; **(B)** The neutral community model (NCM) of community assembly for EM fungal community.

**Figure 6:**
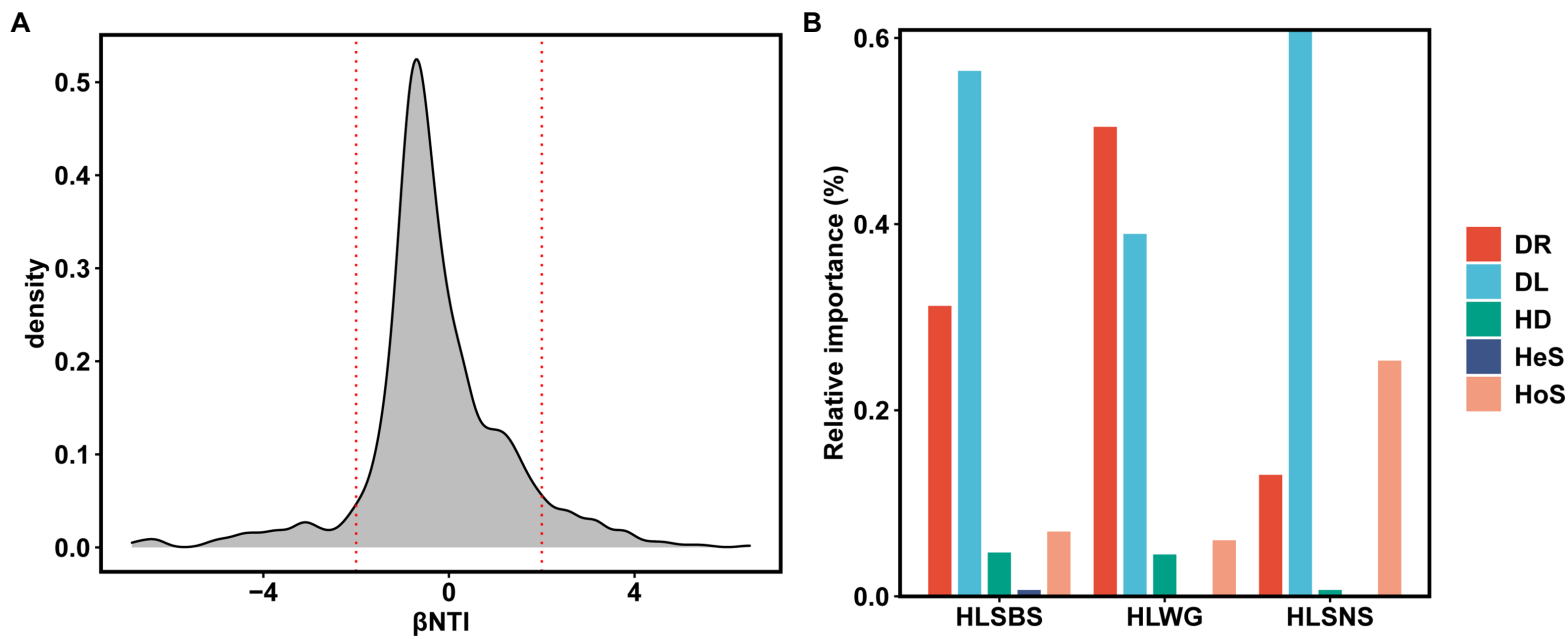
Ecological processes underlying community assembly of ectomycorrhizal (EM) fungi. **(A)** Beta nearest taxon index (βNTI); **(B)** relative importance of ecological processes in determining EM fungal communities in each site. HoS, homogeneous selection; HeS, heterogeneous selection; HD, homogenizing dispersal; DL, dispersal limitation; and DR: drift and others.

Approximately 24.70% (41 out of 166) of OTUs were present in more than 50% of the samples. There was a significant positive relationship between relative abundance and sites occupied by each OTUs (*R* = 0.63, *p* < 0.05, Figure 5A), indicating that rare taxa tended to have a weak ability to disperse and/or adapt (Liu et al., 2020). In addition, we fitted the EM fungal communities to the NCM (Figure 5B). The goodness of fit was explained with 30.4% for EM fungal. EM fungal communities (*m* = 0.007) exhibited a low mitigation rate, indicating that they were mainly limited by dispersal. The βNTI values were used to assess the different ecological assembly processes in EM fungal communities (Figure 6A). Most of the βNTI values of the *P. crassifolia* EM fungal community ranged from −2 to 2. Overall, these results indicated that stochastic processes dominated the assembly of the *P. crassifolia* EM fungal community.

Furthermore, we used the relative importance values of iCAMP to quantify the process of the *P. crassifolia* EM fungal community in the three sites (Figure 6B). The results showed that heterogeneous selection, homogeneous selection, dispersal limitations, homogenizing dispersal, and drift explained 0.677, 6.952, 56.455, 4.707, and 31.209% of the total variability in HLSBS; 0.075, 25.319, 60.864, 0.685, and 13.057% in HLSNS; and 0.088, 6.020, 38.945, 4.498, and 50.449% in HLWG. The results implied that dispersal limitation played the most important role in controlling EM fungal communities in HLSBS and HLSNS, followed by drift and homogeneous selection, while drift was the most important ecological process in driving EM fungal community in HLWG, followed by dispersal limitation. After removing the EM fungal OTUs with the significant site preference, the reanalysis of iCAMP indicated that stochastic processes (drift and dispersal limitation) dominantly predicted the community assembly of EM fungi (Supplementary Figure 6).

## Discussion

Our finding suggested that a high diversity of EM fungi is associated with *P. crassifolia* in this study. Similar but relatively lower fungal diversity of EM fungi in the study that also involved in single EM host plant has been reported by Wang et al. (2021a), in which 122 EM fungal OTUs with richness 13.1 ± 2.2 (mean ± SE) were found in 21 root samples of *Larix gemelinii*. In contrast, Matsuoka et al. (2019) investigated 240 blocks of *Castanopsis sieboldii* and 365 EM fungal OTUs were found. It has been widely accepted that sample size could affect EM fungal diversity in the studies; indeed, our rarefication curves did not reach a plateau, which implied more field work, and samples could bring more undiscovered fungal OTUs. Additionally, the fungal diversity of EM fungi was also affected by the host plant, soil, and climatic variables, which have been reported in many studies conducted at various habitats (Tedersoo et al., 2013; Miyamoto et al., 2018; Wang et al., 2021a). In our study, EM fungal OTUs richness was significantly different across the three sites, and this may be due to the variations in soil and climatic conditions. Previous studies indicated that warming could decrease EM fungal diversity, and accordingly, the MAT in HLSNS and HLSBS was lower than that in HLWG, which may contribute to a relatively higher fungal diversity in the two sites than in HLWG. Additionally, we suspect that proper precipitation could also support the growth of fungi in the semi-arid forests as HLSNS and HLSBS harbored more MAP than HLWG. The soil variables were not recorded in our study, but their influence on EM fungal diversity has been intensively investigated in many previous studies (Lilleskov et al., 2004; Glassman et al., 2015; Miyamoto et al., 2015; Wu et al., 2018; Otsing et al., 2021).

*Sebacina*, *Tomentella-Thelephora*, *Wilcoxina*, and *Amphinema-Tylospora* were the most dominant EM fungal lineages of *P.crassifolia* in the Helan Mountains (accounting for 73.786% of total reads), with *Sebacina* being the most dominant (accounting for 40.828% of total reads). Recent studies have reported that EM fungal community associated with *Larix cajanderi* in eastern Siberia was characterized by a high proportion of *Suillus-Rhizopogon* (Lilleskov et al., 2004; Glassman et al., 2015; Miyamoto et al., 2015; Wu et al., 2018; Otsing et al., 2021), and *Tricholoma* was the most dominant lineage on *Larix gemelinii* in the Great Khingan Mountains, Inner Mongolia, China (Wang et al., 2021b). Obviously, these studies and our finding showed inconsistency with previous studies performed in various forests and host plants to a certain extent, in which *Tomentella-Thelephora*, *Sebacina*, *Inocybe*, and *Russula-Lactarius* were commonly the dominant EM fungal lineages (e.g., Tedersoo et al., 2010; Wu et al., 2018; Wang et al., 2019). Based on the data of abundant EM fungal lineages mentioned above, we found that *Tomentella-Thelephora* was commonly the abundant lineage in many studies, which showed consistency in EM fungal lineages across studies. However, *Tomentella-Thelephora* was not always ranked at the number one in abundance, and other lineages, such as *Sebacina*, *Tricholoma*, and *Suillus-Rhizopogon* could be the most abundant ones, which indicated inconsistency or unique characteristic of each study. This further indicated that some unstudied EM plants and habitats may host distinct EM fungal groups and communities, which may be due to the host effect or local fungal species pool, and this speculation needs to be confirmed in further study.

Thus, we can speculate that the variation in the distribution and/or preference for the site of EM fungal OTUs contributes to the difference in EM fungal communities among the three sites in present study. Additionally, the variations in EM fungal communities and distributions/preference of EM fungal OTUs could be fundamentally attributed to the niche difference among fungal OTUs, because different OTUs preferred various habitats in terms of temperature, soil water, and nutrients (e.g., Glassman et al., 2017; Miyamoto et al., 2018); for example, some EM fungal OTUs could adapt well to relative cold environments, while some preferred warm habitats (Miyamoto et al., 2018). Indeed, in our study, the climatic conditions were different across the three sites, and the climatic condition of HLWG is relatively warmer and moist than HLSNS and HLSBS. Additionally, the difference in climatic conditions could result in distinct soil nutrients, which further drive EM fungal community variations. Although soil data were not available in our study, the significant influence of soil parameters on EM fungal communities has been intensively explored in previous studies as different niche requirements for various fungal taxa (Glassman et al., 2017; Wang et al., 2021a,b). Thus, we suspect that the difference in environmental conditions among the three sampling sites could bring various living niches for EM fungi, and different EM fungi lived in proper habitats for their living, hence resulting in significant variations in EM fungal communities across the three sites. Site/fungus preference indicated that the site harbored site-preference OTUs, and some OTUs showed site preference and preferred certain sites. On the one hand, we suspect that the spatial distance strongly limited the dispersal of these EM fungi between sites (species pool) and, thus, was limited in certain sites (Peay and Bruns, 2014). On the other hand, these fungal OTUs may disperse successfully but with narrow niches and, thus, cannot be successfully colonized in the new habitats (Miyamoto et al., 2018). In summary, the results of preference analysis reflected the significant co-occurrence of EM fungi with the site and, thus, contributed to the variations in EM fungal communities among the three sites.

Overall, stochastic processes (74.606–93.891%) played a more important role than deterministic process (6.109–25.394%) in controlling community assembly of EM fungi associated with *P. crassifolia* in Helan Mountains, in which dispersal limitations (38.945–60.864%) and drift (13.057–50.449%) were most important ecological processes (Figure 6B). The scale of investigations is known to potentially influence the importance of stochastic and deterministic processes in shaping microbial communities (Sun et al., 2021). The lack of significant deterministic processes including homogeneous selection and heterogeneous selection may be due to the fact that this study was conducted at a narrow scale (several km, local scale), and the heterogeneities of environmental conditions may be at low levels (Ning et al., 2020; Sun et al., 2021). A growing body of evidence suggests that the dispersal limitation in stochastic processes has been widely accepted in driving community assembly of soil and root microbes (Beck et al., 2015; Glassman et al., 2015; Wang et al., 2019). The dispersal limitation is derived from the limits of spatial distance on the movement of microbes, thus resulting in a low migration rate of microbes and heterogeneous distribution of microbial community along a spatial distance (Chase, 2003; Segre et al., 2014; Catano et al., 2017; Evans et al., 2017; Ning et al., 2020). Correspondingly, the *m*-value is 0.007 in our study, which suggested a very low dispersal of EM fungi in present study and supported a strong effect of dispersal limitation on community assembly of EM fungi. Drift mirrors the fluctuation of relative abundances of different fungal taxa within a community over time due to the inherently random process of birth, death, and reproduction (Zhou and Ning, 2017). Interestingly, we found a relatively important role of drift than the other ecological processes at the HLWG site, which may be due to the fact that HLWG harbored a relatively lower size of fungal community when compared with those in the other two study sites, as Gao et al. (2020) suggested that stochastic processes were negatively correlated with fungal community size. That is, the smaller the community, the greater effect of the drift. Deterministic processes including heterogeneous and homogeneous selections are defined as ecological forces that drive community composition to become more dissimilar or similar due to fitness differences among different fungal taxa (Zhou and Ning, 2017). In our study, homogeneous selection played a more important role than heterogeneous selection, and this may be caused by homogeneous environments at small spatial scales in our study in comparison with broad spatial scales. Homogeneous selection played a more role in HLSNS than that in HLSBS and HLWG, and we suspected that this may be caused by more even environmental conditions in site HLSBS, although soil data were unavailable in our study. After removing the site-preference OTUs, the ecological processes underlying community assembly changed to some extent, indicating these OTUs with significant preference influenced community assembly, but the stochastic process always dominantly determined community assembly of EM fungi in the present study, and the underlying mechanisms need further investigation in the future study.

## Conclusion

Our study revealed that fungal diversity and ecological processes underlying the community assembly of EM fungi are associated with *P. crassifolia* for the first time. We found a high diversity of EM fungi on *P. crassifolia*, that is, 166 OTUs were identified as EM fungi, belonging to 24 lineages. There were obvious differences in EM fungal communities among the three sampling sites, and some abundant EM fungal OTUs preferred specific sites. The EM fungal community assembly was determined by a combination of deterministic and stochastic processes, but dispersal limitation and drift in the stochastic processes were the predominant processes. These findings improved our understanding of EM fungal diversity and ecological processes of EM fungal communities associated with a single host plant in a semi-arid forest.

## Data availability statement

The datasets presented in this study can be found in online repositories. The names of the repository/repositories and accession number(s) can be found in the article/Supplementary material.

## Author contributions

YF and YW conceived and designed this experiment. JM, SX, YS, and YX collected samples and conducted experiments. XZ participated in the acquisition and analysis of the data. XZ and YW wrote the manuscript. JM and YX participated in the discussion draft of the manuscript. YW, BB, and YF revised the final manuscript. All authors contributed to the article and approved the submitted version.

## Funding

This study was funded by the National Natural Science Foundation of China (nos. 32260006 and 32260027), Inner Mongolia Natural Science Foundation (nos. 2020MS03001 and 2021BS03027), Science and Technology Project of Inner Mongolia Autonomous Region (no. 2019GG002), and the Youth Science and Technology Talent Support Scheme of Colleges and Universities in Inner Mongolia Autonomous Region (no. NJYT-18-A21).

## Conflict of interest

The authors declare that the research was conducted in the absence of any commercial or financial relationships that could be construed as a potential conflict of interest.

## Publisher’s note

All claims expressed in this article are solely those of the authors and do not necessarily represent those of their affiliated organizations, or those of the publisher, the editors and the reviewers. Any product that may be evaluated in this article, or claim that may be made by its manufacturer, is not guaranteed or endorsed by the publisher.
