# Supplementary material for: Stochastic processes dominate community assembly of ectomycorrhizal fungi associated with Picea crassifolia in the Helan Mountains, China
Source: Front Microbiol. 2023 Jan 12;13:1061819. doi: 10.3389/fmicb.2022.1061819 (PMC9878330; doi:10.3389/fmicb.2022.1061819)
Supplement: Supplementary file 1 [file Data_Sheet_1.docx]

**Supplementary Material**

**Supplementary Method**

Detailed description of Site/Fungus preference analysis

**Supplementary Tables**

Supplementary Table 1-3

**Supplementary Figures**

Supplementary Figure 1-6

**Supplementary Codes**

R codes used in the present study

**Supplementary Method**

**Detailed description of Site/Fungus preference analysis**

This analysis was mainly based on the protocol proposed by Toju et al., (2017). Briefly, the sample (row) × EM fungal OTU (column) data matrix was binarized to a sample-level matrix (presence/absence data) and then converted into a site-level matrix (quantitative data), in which rows represent sites, columns represent EM fungal OTUs, and cell entries were the number of samples in which particular Site-OTU combinations were observed.

To perform a randomization analysis, a randomized site-level matrix was generated by shuffling the site labels in the sample-level matrix with 1000 permutations. The *d'* interaction specialization index (Blüthgen *et al*. 2007) was calculated using the dfun function in the bipartite package version 2.05 (Dormann *et al*. 2009). The *d'* value for each site was standardized as follows: Standardized *d'* = [*d'*_observed_ – Mean (*d'*_randomized_)]/SD (*d'*_randomized_), where *d'*_observed_ is the *d'* estimate for the original data, and Mean (*d'*_randomized_) and SD (*d'*_randomized_) are the mean and standard deviation of the *d'* values for the randomized data matrices. The standardized *d'* for each of the fungal OTUs was also calculated based on the original and randomized data matrices as described above. As it is difficult to estimate the site preferences of rare bacteria, we only show the abundant EM fungal OTUs (> 0.5% of total EM fungal sequences). In addition, based on the site-level original and randomized matrices, we evaluated the extent to which each pair of site (i) and fungal OTU (j) was observed (counts) more or less frequently than would be expected by chance. The two-dimensional preferences (*2DP*) were quantified as: *2DP* (i, j) = [*N*_observed_ (i, j) – Mean (*N*_randomized_ (i, j))]/SD (*N*_randomized_ (i, j)), where *N*_observed_ (i, j) is the number of samples from which a pair of a site and a fungal OTU was observed in the original data, and Mean (*N*_randomized_ (i, j)) and SD (*N*_randomized_ (i, j)) are the mean and the standard deviation of the number of samples for the focal site-fungal OTU pair across randomized matrices. The *P* value obtained from the preference analysis was generated by one-tail method and adjusted based on the false discovery rate (FDR; Benjamini and Hochberg 1995).

**References**

Benjamini Y, Hochberg Y. Controlling the false discovery rate: a practical and powerful approach to multiple testing. J R Stat Soc B Met 1995;57:289–300

Blüthgen N, Menzel F, Hovestadt T et al. Specialization, constraints, and conflicting interests in mutualistic networks. Curr Biol 2007;17:341–6.

Dormann CF, Fründ J, Blüthgen N et al. Indices, graphs and null models: analyzing bipartite ecological networks. The Open Ecol J 2009;2:7–24.

Toju H, Tanabe AS, Ishii HS. Ericaceous plant-fungus network in a harsh alpine-subalpine environment. Mol Ecol 2016;25:3242–57.

**Supplementary Tables**

**Table S1.** Information on geographic, climatic parameters and DBH of each site in this study.

| Site | Latitude | Longitude | Altitude | MAT | MAP | DBH |
| --- | --- | --- | --- | --- | --- | --- |
| HLSBS | 38.86 | 105.92 | 2448 | -0.30 | 263 | 17.03±0.24 |
| HLSNS | 38.68 | 105.84 | 2434 | 1.83 | 257 | 16.93±0.45 |
| HLWG | 38.84 | 105.70 | 2444 | 7.50 | 208 | 17.05±0.33 |

HLSBS: HeLanshan-Beisi; HLWG: HaLawugo; HLSNS: HeLanshan-Nansi; MAT, mean annual temperature; MAP, mean annual precipitation; DBH: Diameter at breast height.

**Table S2.** Molecular identification of ectomycorrhizal (EM) fungi in this study. See excel file (Supplementary_Materials_Table_S2).

**Table S3.** The data used for analysis in the present study. See excel file (Supplementary_Materials_Table_S3).

**Supplementary Figures**

**Supplementary Figure 1-6**


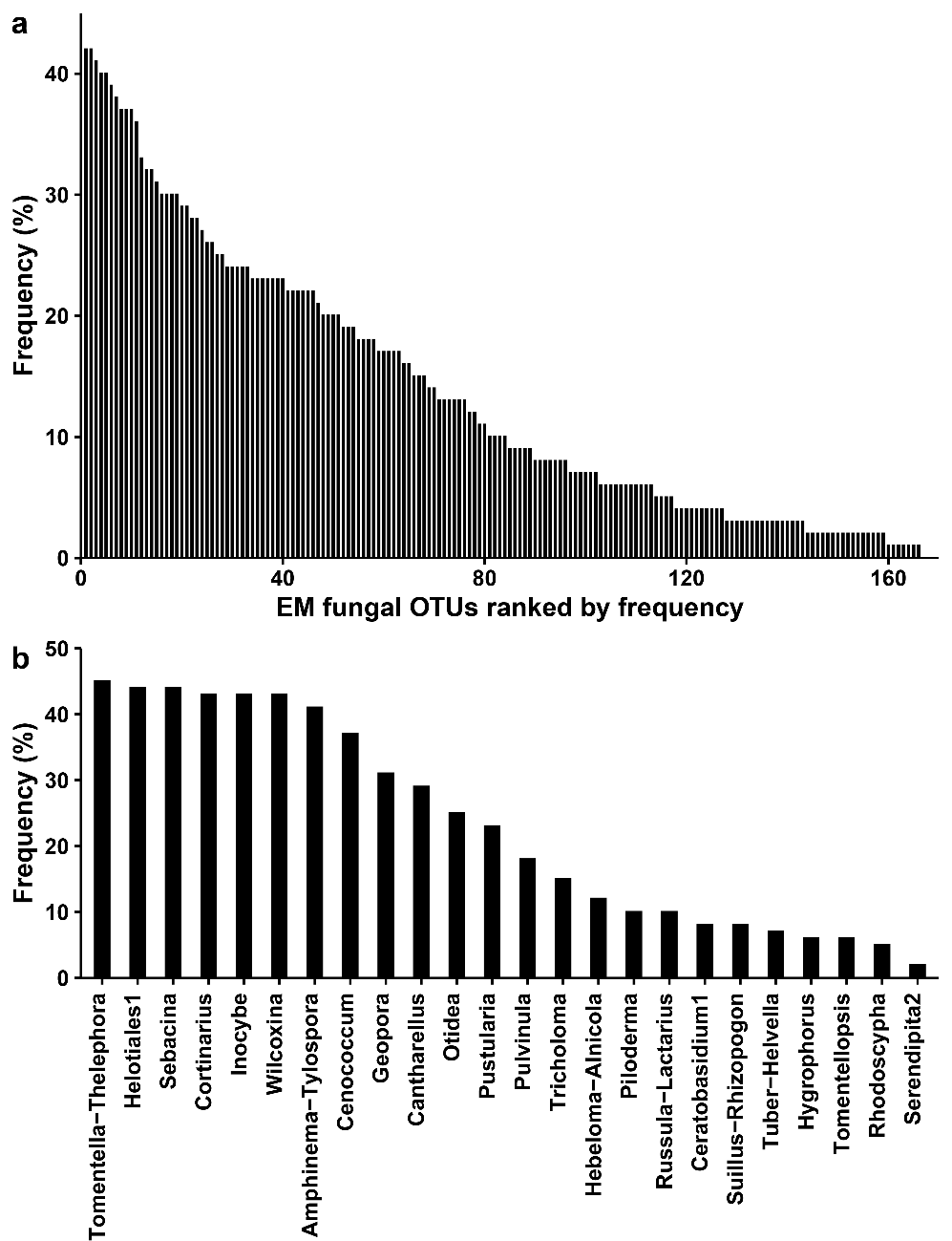


**Supplementary Figure 1.** Ectomycorrhizal (EM) fungal operational taxonomic units (OTUs) ranked by frequency (a) and EM fungal lineages ranked by frequency (b).


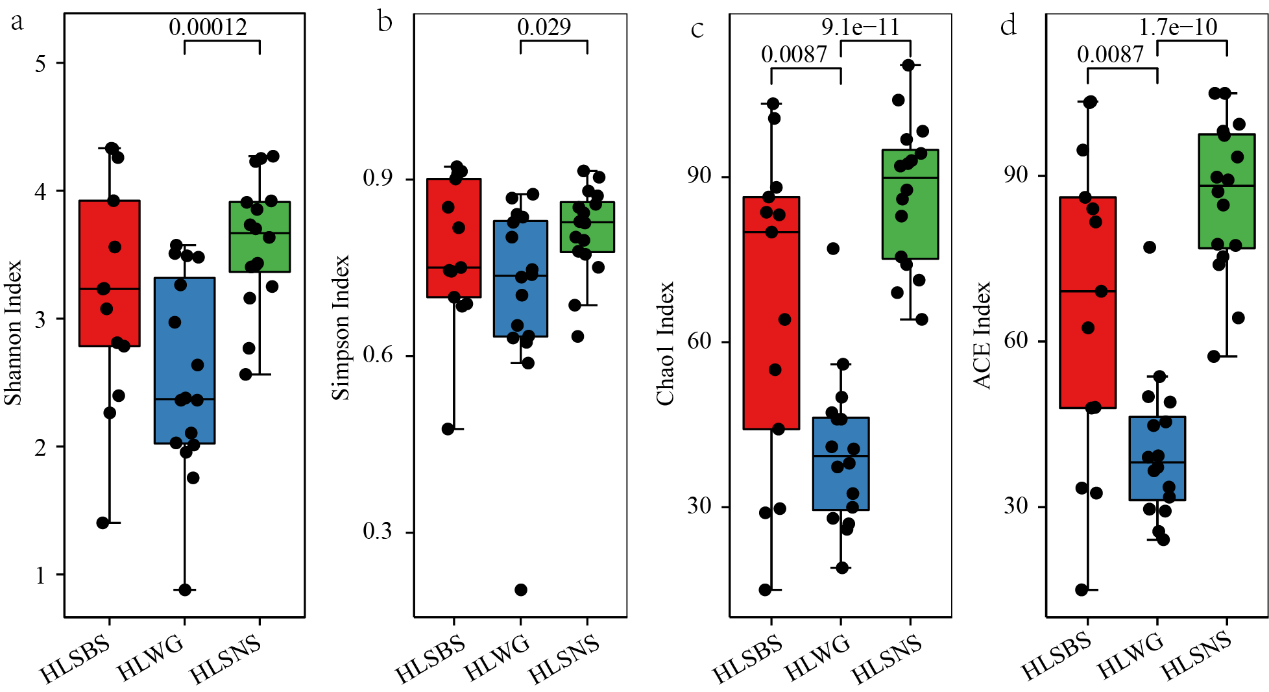


**Supplementary Figure 2.** Differences in diversity index of ectomycorrhizal fungi among the three sites. (a) Shannon index; (b) Simpson index; (c) Chao1 index and (d) ACE index. HLSBS: HeLanshan-Beisi; HLWG: HaLawugo; HLSNS: HeLanshan-Nansi.


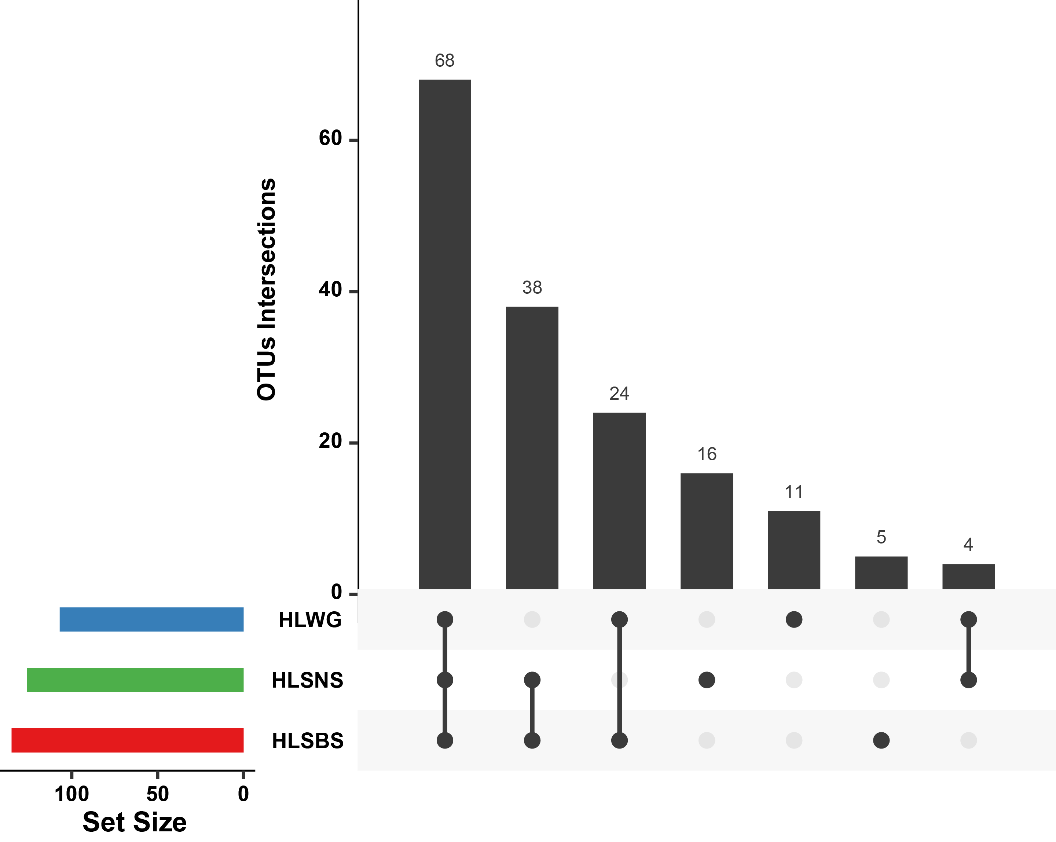


**Supplementary Figure 3.** The numerical relationships of ECM fungal OTUs between HLSBS, HLWG and HLSNS. HLSBS: HeLanshan-Beisi; HLWG: HaLawugo; HLSNS: HeLanshan-Nansi.

**
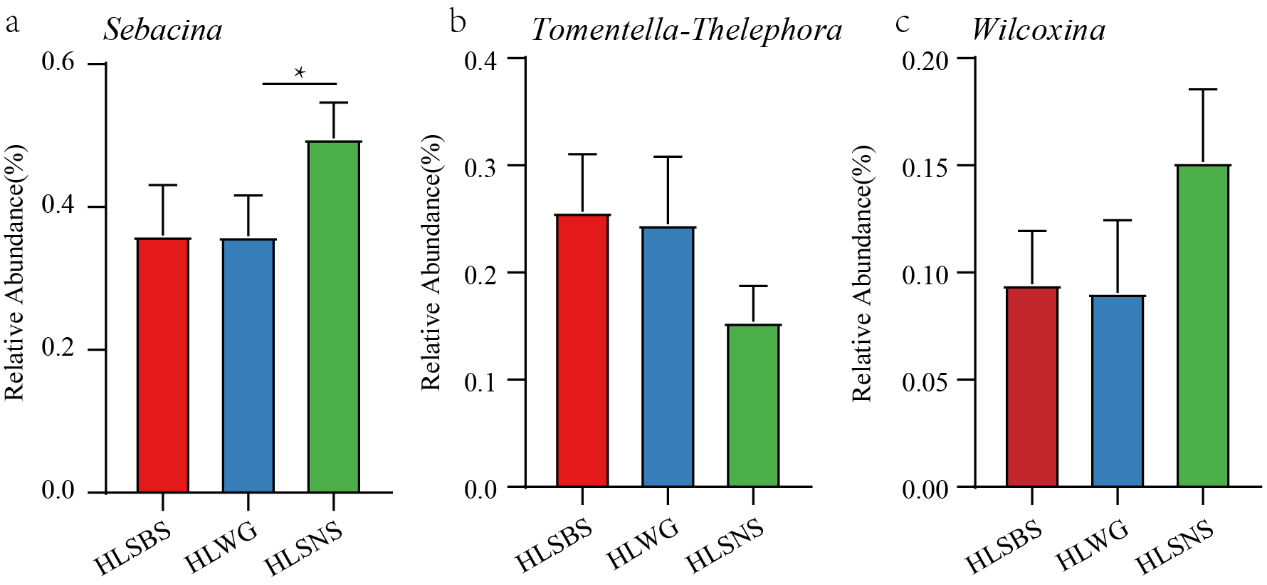
**

**Supplementary Figure 4.** Relative abundances of the three most dominant lineages across sites. (a) *Sebacina*; (b) *Tomentella-Thelephora* and (c) *Wilcoxina*. HLSBS: HeLanshan-Beisi; HLWG: HaLawugo; HLSNS: HeLanshan-Nansi.


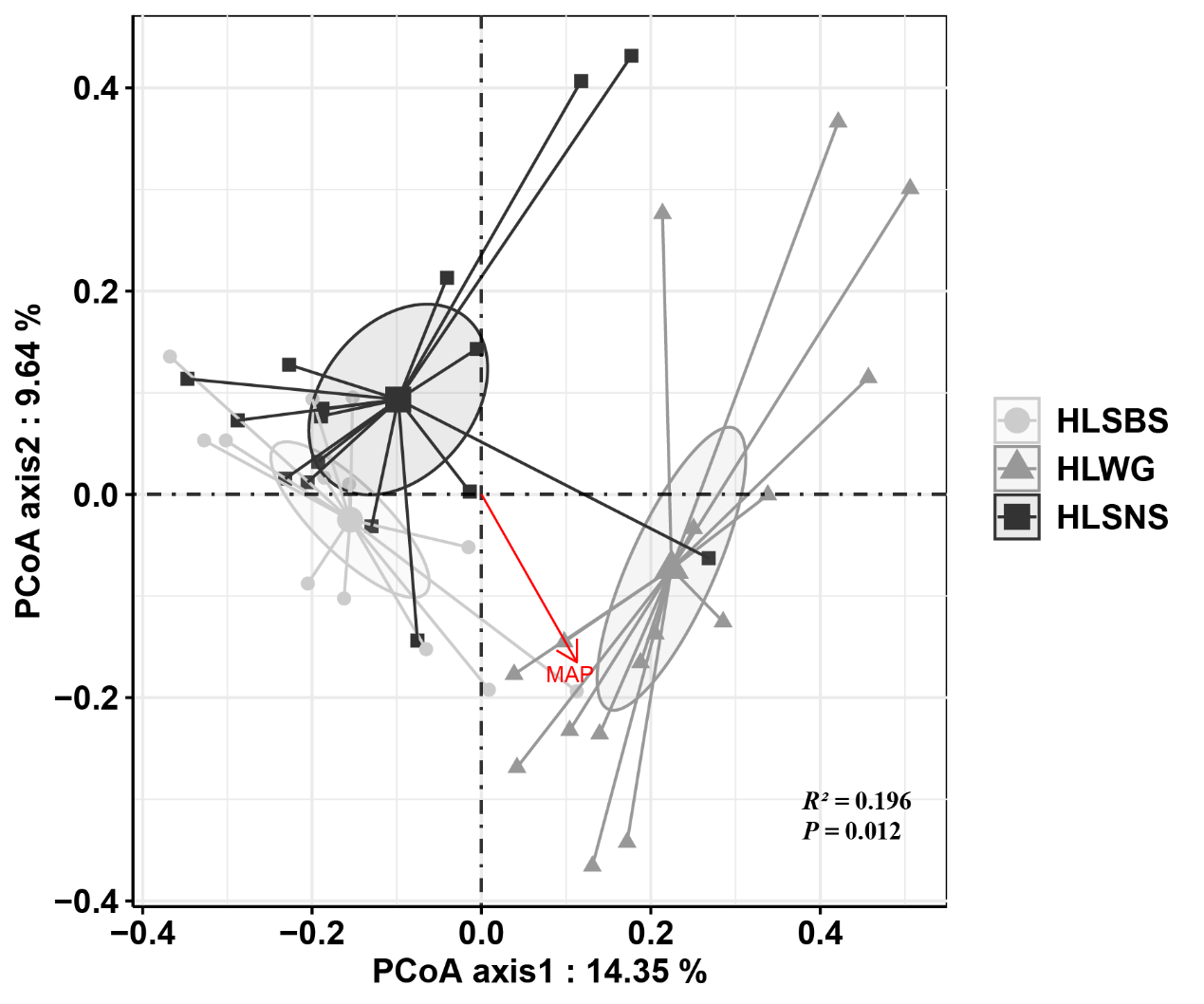


**Supplementary Figure 5.** Environment fitting test revealed significant variables for ectomycorrhizal fungal community composition. MAP, mean annual precipitation; HLSBS: HeLanShan-BeiSi; HLWG: HaLaWuGo; HLSNS: HeLanShan-NanSi.


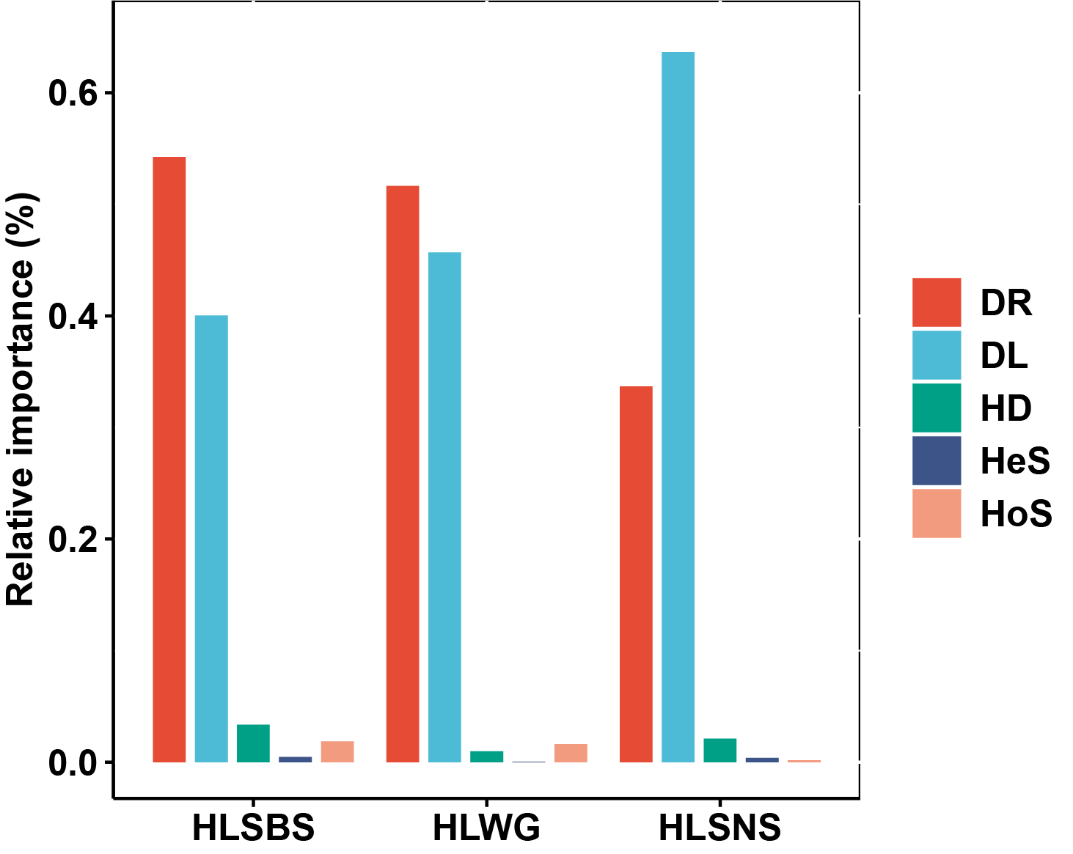


**Supplementary Figure 6.** Relative importance of ecological processes in determining ectomycorrhizal fungal communities in each site; HoS, homogeneous selection; HeS, heterogeneous selection; HD, homogenizing dispersal; DL, dispersal limitation; DR: drift and others.

**Supplementary Codes**

**R codes used in the present study**

#**********************************************************************************************************

######################### Figure1 analysis ###################################

##########################################################################

setwd("/Data")

library(vegan)

library(permute)

library(lattice)

####Figure 1A#######

###Rarefaction of ectomycorrhizal fungal OTUs########

fung<-read.csv("OTU_table and taxonomy.csv",row.names=1,head=T)

fung<-data.frame(decostand(t(fung[,c(-1:-12)]),"hel")) ###"pa" hellinger transformation####

env<-read.csv("group.csv",row.names=1,head=T)

plot(specaccum(fung[env$Sites=="HLWG",][,colSums(fung[env$Sites=="HLWG",])!=0]) ,col="red",xlab="Number of samples", ylab="Number of OTUs",ci=0,lwd=2)

plot(specaccum(fung[env$Sites=="HLSBS",][,colSums(fung[env$Sites=="HLSBS",])!=0]) ,col="blue",xlab="Number of samples", ylab="Number of OTUs",ci=0,lwd=2,add=TRUE)

plot(specaccum(fung[env$Sites=="HLSNS",][,colSums(fung[env$Sites=="HLSNS",])!=0]) ,col="green",xlab="Number of samples", ylab="Number of OTUs",ci=0,lwd=2,add=TRUE)

legend('bottomright', c("HLWG","HLSBS","HLSNS"),

col=c('red',"blue","green"), lty=1,cex=1,lwd=c(2,2,2,2),box.lty=0)

fung0 <-read.csv("OTU_table and taxonomy.csv",row.names=1,head=T)

fung<-data.frame(fung0[,c(-1:-12)])

otus <-data.frame(t(fung))

# ######################################################

# # Alpha Diversity

library(vegan)

alpha <- function(otus, base = exp(1)) {

est <- estimateR(otus)

Richness <- est[1, ]

Chao1 <- est[2, ]

ACE <- est[4, ]

Shannon <- diversity(otus, index = 'shannon', base = base)

Simpson <- diversity(otus, index = 'simpson')

Pielou <- Shannon / log(Richness, base)

goods_coverage <- 1 - rowSums(otus == 1) / rowSums(otus)

result <- data.frame(Richness, Shannon, Simpson, Pielou, Chao1, ACE, goods_coverage)}

alpha_all<- alpha(otus, base = 2)

group<- read.csv("group.csv",header = T,row.names = 1)

alpha.group <- cbind(group, alpha_all[match(rownames(group), rownames(alpha_all)), ])

alpha.group$Sites<-factor(alpha.group$Sites,levels = c("HLSBS","HLWG","HLSNS"))

library(ggpubr)

####Figure 1B#######

Richness=compare_means(Richness~Sites, method = "wilcox.test",data=alpha.group)

Richness

#write.csv(Richness,"Richness_compare.csv")

data1<-list(c("HLSBS","HLWG"),c("HLSBS","HLSNS"),c("HLWG","HLSNS"))

p1<-ggboxplot(data =alpha.group,x="Sites",y="Richness", fill="Sites",main="",notch =FALSE,add = "jitter",palette = "Set1",bxp.errorbar = T,bxp.errorbar.width = 0.3, width = 0.8)+labs(x = '', y = 'Richness Index')+stat_compare_means(method="t.test",comparisons =data1,label="p.format",label.y = c(80,90,85))

p1

ggpar(p1,legend = "right", legend.title = "",font.legend = c(10))

p4<-p1+theme(panel.background = element_rect(fill='white', colour='black'),

panel.border = element_blank(),

title = element_text(face = 'bold',size=15),

axis.line = element_line(colour = "black"),

axis.title = element_text(face = 'bold',size=12),

axis.ticks = element_line(color='black'),

axis.title.x=element_text(colour='black', size=12),

axis.title.y=element_text(face = 'bold', size=12),

axis.text=element_text(colour='black',size=12),

legend.title=element_blank(),

legend.text=element_text(face = 'bold',size=12),

legend.key=element_blank(),legend.position = c(),

axis.text.y = element_text(face = 'bold',size=12),

axis.text.x = element_text(face = 'bold',size=12))

p4

##

###########Supplementary Figure 2a#####################################

library(vegan)

library(ggpubr)

Shannon=compare_means(Shannon~Sites, method = "wilcox.test",data=alpha.group)

Shannon

write.csv(Shannon,"Shannon_compare.csv")

data1<-list(c("HLWG","HLSNS"))

p1<-ggboxplot(data =alpha.group,x="Sites",y="Shannon", fill="Sites",main="",notch =FALSE,add = "jitter",palette = "Set1",bxp.errorbar = T,bxp.errorbar.width = 0.3, width = 0.8)+labs(x = '', y = 'Shannon Index')+stat_compare_means(method="wilcox.test",comparisons =data1,label="p.format",label.y = c(5))

p1

ggpar(p1,legend = "right", legend.title = "",font.legend = c(10))

p4<-p1+theme(panel.background = element_rect(fill='white', colour='black'),

panel.border = element_blank(),

title = element_text(face = 'bold',size=15),

axis.line = element_line(colour = "black"),

axis.title = element_text(face = 'bold',size=12),

axis.ticks = element_line(color='black'),

axis.title.x=element_text(colour='black', size=12),

axis.title.y=element_text(face = 'bold', size=12),

axis.text=element_text(colour='black',size=12),

legend.title=element_blank(),

legend.text=element_text(face = 'bold',size=12),

legend.key=element_blank(),legend.position = c(),

axis.text.y = element_text(face = 'bold',size=12),

axis.text.x = element_text(face = 'bold',size=12))

p4

##

###########Supplementary Figure 2b#####################################

Simpson=compare_means(Simpson~Sites, method = "wilcox.test",data=alpha.group)

Simpson

write.csv(Simpson,"Simpson_compare.csv")

data1<-list(c("HLWG","HLSNS"))

p1<-ggboxplot(data =alpha.group,x="Sites",y="Simpson", fill="Sites",main="",notch =FALSE,add = "jitter",palette = "Set1",bxp.errorbar = T,bxp.errorbar.width = 0.3, width = 0.8)+labs(x = '', y = 'Simpson Index')+stat_compare_means(method="wilcox.test",comparisons =data1,label="p.format",label.y = c(1.1))

p1

ggpar(p1,legend = "right", legend.title = "",font.legend = c(10))

p4<-p1+theme(panel.background = element_rect(fill='white', colour='black'),

panel.border = element_blank(),

title = element_text(face = 'bold',size=15),

axis.line = element_line(colour = "black"),

axis.title = element_text(face = 'bold',size=12),

axis.ticks = element_line(color='black'),

axis.title.x=element_text(colour='black', size=12),

axis.title.y=element_text(face = 'bold', size=12),

axis.text=element_text(colour='black',size=12),

legend.title=element_blank(),

legend.text=element_text(face = 'bold',size=12),

legend.key=element_blank(),legend.position = c(),

axis.text.y = element_text(face = 'bold',size=12),

axis.text.x = element_text(face = 'bold',size=12))

p4

##

###########Supplementary Figure 2c#########################

Chao1=compare_means(Chao1~Sites, method = "wilcox.test",data=alpha.group)

Chao1

write.csv(Chao1,"Chao1_compare.csv")

data1<-list(c("HLWG","HLSNS"),c("HLWG","HLSBS"))

p1<-ggboxplot(data =alpha.group,x="Sites",y="Chao1", fill="Sites",main="",notch =FALSE,add = "jitter",palette = "Set1",bxp.errorbar = T,bxp.errorbar.width = 0.3, width = 0.8)+labs(x = '', y = 'Chao1 Index')+stat_compare_means(method="wilcox.test",comparisons =data1,label="p.format",label.y = c(110,105))

p1

ggpar(p1,legend = "right", legend.title = "",font.legend = c(10))

p4<-p1+theme(panel.background = element_rect(fill='white', colour='black'),

panel.border = element_blank(),

title = element_text(face = 'bold',size=15),

axis.line = element_line(colour = "black"),

axis.title = element_text(face = 'bold',size=12),

axis.ticks = element_line(color='black'),

axis.title.x=element_text(colour='black', size=12),

axis.title.y=element_text(face = 'bold', size=12),

axis.text=element_text(colour='black',size=12),

legend.title=element_blank(),

legend.text=element_text(face = 'bold',size=12),

legend.key=element_blank(),legend.position = c(),

axis.text.y = element_text(face = 'bold',size=12),

axis.text.x = element_text(face = 'bold',size=12))

p4

##

###########Supplementary Figure 2d#####################################

ACE=compare_means(ACE~Sites, method = "wilcox.test",data=alpha.group)

ACE

write.csv(ACE,"ACE_compare.csv")

data1<-list(c("HLWG","HLSNS"),c("HLWG","HLSBS"))

p1<-ggboxplot(data =alpha.group,x="Sites",y="ACE", fill="Sites",main="",notch =FALSE,add = "jitter",palette = "Set1",bxp.errorbar = T,bxp.errorbar.width = 0.3, width = 0.8)+labs(x = '', y = 'ACE Index')+stat_compare_means(method="wilcox.test",comparisons =data1,label="p.format",label.y = c(110,105))

p1

ggpar(p1,legend = "right", legend.title = "",font.legend = c(10))

p4<-p1+theme(panel.background = element_rect(fill='white', colour='black'),

panel.border = element_blank(),

title = element_text(face = 'bold',size=15),

axis.line = element_line(colour = "black"),

axis.title = element_text(face = 'bold',size=12),

axis.ticks = element_line(color='black'),

axis.title.x=element_text(colour='black', size=12),

axis.title.y=element_text(face = 'bold', size=12),

axis.text=element_text(colour='black',size=12),

legend.title=element_blank(),

legend.text=element_text(face = 'bold',size=12),

legend.key=element_blank(),legend.position = c(),

axis.text.y = element_text(face = 'bold',size=12),

axis.text.x = element_text(face = 'bold',size=12))

p4

#**********************************************************************************************************

######################### Figure2 analysis ###################################

##########################################################################setwd("/Data")

library(vegan)

fung0 <-read.csv("OTU_table and taxonomy.csv",row.names=1,head=T)

fung <- data.frame(fung0[,c(-1:-11)])

head(fung)

library(dplyr)

fung1 <- group_by(fung,lineage)%>%summarise_all(funs(sum))

fung1 <- data.frame(fung1)

rownames(fung1)<- fung1[,1]

fung1 <- data.frame(t(fung1[,-1]))

group<-read.csv("group.csv",row.names = 1,head=T)

ab=cbind(group,fung1[match(rownames(group),rownames(fung1)), ])

ab1= group_by(ab,Sites)%>%summarise_all(funs(mean))

ab1 <- data.frame(ab1)

rownames(ab1)<- ab1[,1]

ab1 <- data.frame(t(ab1[,-1]))

df_ab<- matrix(0,nrow = nrow(ab1), ncol = ncol(ab1))

for(i in 1:ncol(ab1)){

df_ab[,i] = ab1[ ,i]/sum(ab1[ ,i])

}

colnames(df_ab) <- colnames(ab1)

rownames(df_ab) <- rownames(ab1)

df_ab<-as.matrix(x=df_ab,row.names=1,col.names=1)

df_ab<- as.data.frame(df_ab)

write.csv(df_ab,"lineage_ab.csv")

df_ab$sum <- rowSums(df_ab)

df_ab <- df_ab[order(df_ab$sum, decreasing = TRUE), ]

df_ab <- df_ab[1:15, -ncol(df_ab)]

df_ab['Others', ] <- 1 - colSums(df_ab)

df_ab$Lineage <- factor(rownames(df_ab), levels = rev(rownames(df_ab)))

library(reshape2)

df_ab<- melt(df_ab, id = 'Lineage')

head(df_ab)

library(ggalluvial)

library(ggplot2)

df_ab$variable <- factor(df_ab$variable,c("HLSBS","HLWG","HLSNS"))

p <- ggplot(df_ab, aes(variable, value*100, fill = Lineage)) +

geom_col(position = 'stack', width = 0.5)+labs(x = '', y = 'Relative Abundance(%)')+scale_fill_manual(values = rev(c('#8DD3C7', '#FFFFB3', '#BEBADA', '#FB8072', '#80B1D3', '#FDB462', '#B3DE69', '#FCCDE5', '#BC80BD','#99E8A1',"#17BDCB","#04D0C4","#A68784","#C1D3B9","#16F8D3",'gray')))+scale_y_continuous(expand = c(0,0))

p

p4<-p+theme(panel.background = element_rect(fill='white', colour='black'),

title = element_text(face = 'bold',size=15),

axis.line = element_line(colour = "black"),

axis.title = element_text(face = 'bold',size=12),

axis.ticks = element_line(color='black'),

axis.title.x=element_text(colour='black', size=12),

axis.title.y=element_text(face = 'bold', size=12),

axis.text=element_text(colour='black',size=12),

legend.title=element_blank(),

legend.text=element_text(face = 'bold',size=12),

legend.key=element_blank(),legend.position = c(),

axis.text.y = element_text(face = 'bold',size=12),

axis.text.x = element_text(face = 'bold',size=12))

p4

#**********************************************************************************************************

######################### Figure3 analysis ###################################

##########################################################################

setwd("/Data")

###α分析########

library(vegan)

fung0 <-read.csv("OTU_table and taxonomy.csv",row.names=1,head=T)

fung<-data.frame(fung0[,c(-1:-12)])

genus <-data.frame(t(fung))

library(vegan)

distance <- vegdist(genus,"bray")

pcoa <- cmdscale(distance, k=(nrow(genus)-1), eig = TRUE)

pcoa_eig <- (pcoa$eig)[1:2] / sum(pcoa$eig)

pcoa_exp <- pcoa$eig/sum(pcoa$eig)

pcoa1 <- paste('PCoA axis1 :', round(100*pcoa_exp[1], 2), '%')

pcoa2 <- paste('PCoA axis2 :', round(100*pcoa_exp[2], 2), '%')

sample_site <- data.frame({pcoa$point})[1:2]

colnames(sample_site)<- c("PCoA1","PCoA2")

group<- read.csv("group.csv",header = T,row.names = 1)

plotdata_pcoa = cbind(group, sample_site[match(rownames(group), rownames(sample_site)), ])

plotdata_pcoa$Sites <- factor(plotdata_pcoa$Sites,c("HLSBS","HLWG","HLSNS"))

library(ggpubr)

library(ggplot2)

library(ggrepel)

p1 <- ggscatter(plotdata_pcoa, x= "PCoA1", y = "PCoA2", color = "Sites", palette = "Set1",ellipse = T,ellipse.type ="confidence",mean.point = T,star.plot = T, ggtheme = theme_minimal())

p1

p2<- p1+labs(x = paste(pcoa1),y = paste( pcoa2))+geom_hline(yintercept = 0,lty=4,lwd=0.6,alpha=0.8)+geom_vline(xintercept = 0,lty=4,lwd=0.6,alpha=0.8)

p2

adonis_individual <- adonis(genus~Sites, group, distance = 'bray', permutations = 1000)

adonis_individual

p3<-p2+annotate('text', label = '', x = 0.25, y = 0.4, size = 5) +annotate('text', label = sprintf('italic(P) == %.3f', adonis_individual$aov.tab[1,6]), x = 0.25, y = 0.40, size = 5, parse = TRUE)

p3

p4<-p3+theme(panel.background = element_rect(fill='white', colour='black'),

panel.border = element_blank(),

title = element_text(face = 'bold',size=15),

axis.line = element_line(colour = "black"),

axis.title = element_text(face = 'bold',size=12),

axis.ticks = element_line(color='black'),

axis.title.x=element_text(colour='black', size=12),

axis.title.y=element_text(face = 'bold', size=12),

axis.text=element_text(colour='black',size=12),

legend.title=element_blank(),

legend.text=element_text(face = 'bold',size=12),

legend.key=element_blank(),legend.position = c(),

axis.text.y = element_text(face = 'bold',size=12),

axis.text.x = element_text(face = 'bold',size=12))

p4

#**********************************************************************************************************

######################### Figure4 analysis ###################################

###################################################################################From_Dr.Toju##############

library(bipartite)

library(parallel)

n.core <- 1

nrep <- 1000

setwd("/Data")

xx <- read.table("otu.group.txt", header=T)

xx[xx>0]=1

row.sample <- list(NULL)

for (i in 1:nrep) { row.sample[[i]] <- sample(xx[, 1]) }

gc()

gc()

null.matrices <- list(NULL)

for (i in 1:nrep) { null.matrices[[i]] <- cbind(row.sample[[i]], xx[, -1]) }

gc()

gc()

species.level <- list(NULL)

plant <- unique(xx[, 1])

for (j in 1:nrep) {

species.level[[j]] <- matrix(NA, nrow=length(plant), ncol=ncol(xx[, -1]))

for (i in 1:length(plant)) {

species.level[[j]][i, ] <- colSums(subset(null.matrices[[j]], null.matrices[[j]][, 1]==plant[i])[, -1])

}}

gc()

gc()

m.null <- c(NULL)

n.null <- c(NULL)

for (i in 1:nrep) {

m.null[i] <- ncol(species.level[[i]])

n.null[i] <- nrow(species.level[[i]])

}

gc()

gc()

# original matrix

original <- matrix(NA, nrow=length(plant), ncol=ncol(xx[, -1]))

for (i in 1:length(plant)) {

original[i, ] <- colSums(subset(xx, xx[, 1]==plant[i])[, -1])

}

plant.label <- as.vector(plant)

rownames(original) <- plant.label

colnames(original) <- colnames(xx[, -1])

write.csv(original,"original.csv")

#original

d.plant <- dfun(original)

plant.table <- cbind(unlist(d.plant[[1]]), unlist(d.plant[[2]]), unlist(d.plant[[3]]), unlist(d.plant[[4]]))

colnames(plant.table) <- c("dprime", "d", "dmin", "dmax")

write.table(plant.table, file="dfun.QHYS.plant.txt", quote=F, sep="\t")

d.fungus <- dfun(t(original))

fungus.table <- cbind(unlist(d.fungus[[1]]), unlist(d.fungus[[2]]), unlist(d.fungus[[3]]), unlist(d.fungus[[4]]))

colnames(fungus.table) <- c("dprime", "d", "dmin", "dmax")

write.table(fungus.table, file="dfun.QHYS.fungus.txt", quote=F, sep="\t")

# nulls calc

d.low <- mclapply(species.level, dfun, mc.cores=n.core)

gc()

gc()

species.level.trans <- mclapply(species.level, t, mc.cores=n.core)

gc()

gc()

d.high <- mclapply(species.level.trans, dfun, mc.cores=n.core)

gc()

gc()

d.low.vec <- c(unlist(d.low[[1]][1]))

for (i in 2:nrep) { d.low.vec <- rbind(d.low.vec, c(unlist(d.low[[i]][1])))}

gc()

gc()

d.high.vec <- c(unlist(d.high[[1]][1]))

for (i in 2:nrep) { d.high.vec <- rbind(d.high.vec, c(unlist(d.high[[i]][1])))}

gc()

gc()

colnames(d.low.vec) <- plant.label

rownames(d.low.vec) <- seq(1:nrep)

colnames(d.high.vec) <- colnames(xx[, -1])

rownames(d.high.vec) <- seq(1:nrep)

write.table(round(d.low.vec, 4), file="dfun.plant.rand1000.QHYS.txt", quote=F, row.name=F, sep="\t")

write.table(round(d.high.vec, 4), file="dfun.fungus.rand1000.QHYS.txt", quote=F, row.name=F, sep="\t")

####

plant_mean<-apply(d.low.vec, MARGIN=2,FUN=mean)

plant_sd<-apply(d.low.vec, MARGIN=2,FUN=sd)

plant_mean_sd<-cbind(plant_mean,plant_sd)

write.csv(plant_mean_sd, "dfun.plant_QHYS.mean_sd.csv")

fungus_mean<-apply(d.high.vec, MARGIN=2,FUN=mean)

fungus_sd<-apply(d.high.vec, MARGIN=2,FUN=sd)

fungus_mean_sd<-cbind(fungus_mean,fungus_sd)

write.csv(fungus_mean_sd, "dfun.fungus_QHYS.mean_sd.csv")

mean.n <- matrix(NA, nrow=length(plant), ncol=ncol(xx[, -1]))

sd.n <- matrix(NA, nrow=length(plant), ncol=ncol(xx[, -1]))

for (i in 1:length(plant)) {

for (j in 1:ncol(xx[, -1])) {

n.vec <- c(unlist(species.level[[1]][i,j]))

for (n in 2:nrep) { n.vec <- rbind(n.vec, c(unlist(species.level[[n]][i,j])))}

mean<-apply(n.vec, MARGIN=2,FUN=mean)

sd<-apply(n.vec, MARGIN=2,FUN=sd)

mean.n[i,j]<-mean

sd.n[i,j]<-sd

}}

rownames(mean.n) <- plant.label

colnames(mean.n) <- colnames(xx[, -1])

rownames(sd.n) <- plant.label

colnames(sd.n) <- colnames(xx[, -1])

write.csv(mean.n, "QHYS_mean.csv")

write.csv(sd.n, "QHYS_sd.csv")

###计算得出2DP#####

a<-(original-mean.n)/sd.n

write.csv(a,"2DP_QHYS.csv")

write.csv(original,"original.csv")

##

# z score

randomized.mean <- matrix(NA, nrow=length(plant), ncol=ncol(xx[, -1]))

randomized.sd <- matrix(NA, nrow=length(plant), ncol=ncol(xx[, -1]))

z.score <- matrix(NA, nrow=length(plant), ncol=ncol(xx[, -1]))

vector <- c(NULL)

for (j in 1:ncol(xx[, -1])) {

for (i in 1:length(plant)) {

for (h in 1:nrep) {

vector[h] <- species.level[[h]][i, j]

}

randomized.mean[i, j] <- mean(vector)

}}

gc()

gc()

for (j in 1:ncol(xx[, -1])) {

for (i in 1:length(plant)) {

for (h in 1:nrep) {

vector[h] <- species.level[[h]][i, j]

}

randomized.sd[i, j] <- sd(vector)

}}

gc()

gc()

for (j in 1:ncol(xx[, -1])) {

for (i in 1:length(plant)) {

for (h in 1:nrep) {

vector[h] <- species.level[[h]][i, j]

}

z.score[i, j] <- (original[i, j] - mean(vector)) / sd(vector)

}}

P.value <- matrix(NA, nrow=length(plant), ncol=ncol(xx[, -1]))

for (j in 1:ncol(xx[, -1])) {

for (i in 1:length(plant)) {

for (h in 1:nrep) {

vector[h] <- species.level[[h]][i, j]

}

P.value[i, j] <- sum(vector > original[i, j]) / nrep

}}

gc()

gc()

colnames(original) <- colnames(xx[, -1])

colnames(randomized.mean) <- colnames(xx[, -1])

colnames(randomized.sd) <- colnames(xx[, -1])

colnames(z.score) <- colnames(xx[, -1])

colnames(P.value) <- colnames(xx[, -1])

plant.label <- as.vector(plant)

table.original <- cbind(plant.label, original)

table.randomized.mean <- cbind(plant.label, randomized.mean)

table.randomized.sd <- cbind(plant.label, randomized.sd)

table.z.score <- cbind(plant.label, z.score)

table.P.value <- cbind(plant.label, P.value)

write.table(table.original, file="QHYS.original.txt", sep="\t", quote=F, row.names=F, col.names=T)

write.table(table.randomized.mean, file="QHYS.randomized.mean.txt", sep="\t", quote=F, row.names=F, col.names=T)

write.table(table.randomized.sd , file="QHYS.randomized.sd .txt", sep="\t", quote=F, row.names=F, col.names=T)

write.table(table.z.score, file="QHYS.z.score.txt", sep="\t", quote=F, row.names=F, col.names=T)

write.table(table.P.value, file="QHYS.P.value.txt", sep="\t", quote=F, row.names=F, col.names=T)

#

#save.image("C:/Users/Administrator/Desktop/qhys/QHYS_specificity.Rdata")

###Figure########

library(lattice)

library(RColorBrewer)

library(grid)

xx <- as.matrix(read.table("2DP_QHYS_identy.txt", row.name=1, header=T))

label <- rownames(xx)

pal2 <- colorRampPalette(c("white","pink", "deeppink2", "deeppink4", "black"), space = "rgb")

levelplot(xx, col.regions=(pal2),cuts=15,border="white",colorkey=FALSE)

xx <- as.matrix(read.table("2DP_QHYS_identy.txt", row.name=1, header=T))

label <- rownames(xx)

pal2 <- colorRampPalette(c("white","pink", "deeppink2", "deeppink4", "black"), space = "rgb")

levelplot(xx, col.regions=(pal2),cuts=15,border="white")

xx <- as.matrix(read.table("standard_dprime_sites.txt", row.name=1, header=T))

label <- rownames(xx)

pal2 <- colorRampPalette(c("white","pink", "deeppink2", "deeppink4", "black"), space = "rgb")

levelplot(xx, col.regions=(pal2), cuts=15,border="white")

xx <- as.matrix(read.table("fung_dprime_identy.txt", row.name=1, header=T))

label <- rownames(xx)

pal2 <- colorRampPalette(c("white","pink", "deeppink2", "deeppink4", "black"), space = "rgb")

levelplot(t(xx), col.regions=(pal2),cuts=15,border="white")

#####

###绘制Figure 4b图#####

P <- as.matrix(read.table("QHYS.P.value.txt", row.name=1, header=T))

Z <- as.matrix(read.table("QHYS.z.score.txt", row.names = 1, header=T))

Pvec <- as.vector(P)

Zvec <- as.vector(Z)

fdr <- Pvec

fdr[which(Zvec < 0)] <- 1-fdr[which(Zvec < 0)]

fdr <- p.adjust(fdr, method="fdr")

plot(Zvec, fdr, xlab="Two-dimensional preference (2DP)", ylab="P value (FDR)")

abline(h=0.05, lty=3)

abline(v=1.8, lty=2)

abline(v=-2.5, lty=2)

#######

###adjust p_value based on "fdr" method###

p_fungus <- as.matrix(read.table("p_value_fungus.txt", row.name=1, header=T))

label <- rownames(p_fungus)

p_adj<-p.adjust(p_fungus,method="fdr")

write.csv(p_adj,"p_fungus_fdr.csv")

##

###adjust p_value based on "fdr" method###

p_host <- as.matrix(read.table("p_value_host.txt", row.name=1, header=T))

label <- rownames(p_host)

p_adj<-p.adjust(p_host,method="fdr")

write.csv(p_adj,"p_host_fdr.csv")

###

p_host <- as.matrix(read.table("p_value_host.txt", row.name=1, header=T))

label <- rownames(p_host)

p_adj<-p.adjust(p_host,method="fdr")

write.csv(p_adj,"p_host_fdr.csv")

#**********************************************************************************************************

######################### Figure5 analysis ###################################

##########################################################################

#####Figure5a##################

fung0 <- read.csv("OTU_table and taxonomy.csv",row.names=1,header=T)

fung <- data.frame(fung0[,c(-1:-12)])

fung$average = rowMeans(fung)

df_ab<- matrix(0,nrow = nrow(fung), ncol = ncol(fung))

for(i in 1:ncol(fung)){

df_ab[,i] = fung[ ,i]/sum(fung[ ,i])

}

colnames(df_ab) <- colnames(fung)

rownames(df_ab) <- rownames(fung)

df_ab<-as.matrix(x=df_ab,row.names=1,col.names=1)

df_ab<- as.data.frame(df_ab)

write.csv(df_ab,"abundance.csv")

avg=data.frame(df_ab[,c(-1:-45)])

rownames(avg)=rownames(avg)

colnames(avg)=c("avg")

fung2<-data.frame(fung0[,c(-1:-13)])

fung2[fung2>=1] <- 1

fung2$occurrece=rowSums(fung2)

occ=data.frame(fung2$occurrece)

rownames(occ)=rownames(occ)

colnames(occ)=c("occurrece")

plotdata= cbind(avg, occ[match(rownames(avg), rownames(occ)), ])

colnames(plotdata)=c("avg","occurrece")

library(ggpubr)

library(ggplot2)

library(ggrepel)

library(ggpubr)

p1=ggscatter(plotdata, x = "occurrece", y = "avg",

color = "red", size =2, # Points color and size

add = "reg.line", # Add regression line

add.params = list(color = "blue", fill = "gray"), # Customize regression line

conf.int = TRUE, # Add confidence interval

cor.coef = TRUE, # Add correlation coefficient. see ?stat_cor

cor.coeff.args = list(method = "pearson"))

p2 <- p1+labs(x = "Occurrence",y = "average relative abundance")

p2

p4<-p2+theme(panel.background = element_rect(fill='white', colour='black'),

panel.border = element_blank(),

title = element_text(face = 'bold',size=15),

axis.line = element_line(colour = "black"),

axis.title = element_text(face = 'bold',size=12),

axis.ticks = element_line(color='black'),

axis.title.x=element_text(colour='black', size=12),

axis.title.y=element_text(face = 'bold', size=12),

axis.text=element_text(colour='black',size=12),

legend.title=element_blank(),

legend.text=element_text(face = 'bold',size=12),

legend.key=element_blank(),legend.position = c(),

axis.text.y = element_text(face = 'bold',size=12),

axis.text.x = element_text(face = 'bold',size=12))

p4

#####Figure5b##################

library(vegan)

setwd("/Data")

fung0 <- read.csv("OTU_table and taxonomy.csv",row.names=1,header=T)

fung <- data.frame(fung0[,c(-1:-12)])

library(Hmisc)

library(minpack.lm)

library(stats4)

spp <- data.frame(t(fung))

N <- mean(apply(spp, 1, sum))

p.m <- apply(spp, 2, mean)

p.m <- p.m[p.m != 0]

p <- p.m/N

spp.bi <- 1*(spp>0)

freq <- apply(spp.bi, 2, mean)

freq

freq <- freq[freq != 0]

C <- merge(p, freq, by=0)

C <- C[order(C[,2]),]

C <- as.data.frame(C)

C.0 <- C[!(apply(C, 1, function(y) any(y == 0))),]

p <- C.0[,2]

freq <- C.0[,3]

names(p) <- C.0[,1]

names(freq) <- C.0[,1]

d = 1/N

m.fit <- nlsLM(freq ~ pbeta(d, N*m*p, N*m*(1 -p), lower.tail=FALSE),start=list(m=0.1))

m.fit #获取 m 值

m.ci <- confint(m.fit, 'm', level=0.95)

freq.pred <- pbeta(d, N*coef(m.fit)*p, N*coef(m.fit)*(1 -p), lower.tail=FALSE)

pred.ci <- binconf(freq.pred*nrow(spp), nrow(spp), alpha=0.05, method="wilson", return.df=TRUE)

Rsqr <- 1 - (sum((freq - freq.pred)^2))/(sum((freq - mean(freq))^2))

Rsqr

write.csv(p, file = "p.csv")

write.csv(freq, file = "freq.csv")

write.csv(freq.pred, file = "freq.pred.csv")

bacnlsALL <-data.frame(p,freq,freq.pred,pred.ci[,2:3])

inter.col<-rep('black',nrow(bacnlsALL))

inter.col[bacnlsALL$freq <= bacnlsALL$Lower]<-'#A52A2A'

inter.col[bacnlsALL$freq >= bacnlsALL$Upper]<-'#29A6A6'

library(grid)

grid.newpage()

pushViewport(viewport(h=0.6,w=0.6))

pushViewport(dataViewport(xData=range(log10(bacnlsALL$p)), yData=c(0,1.02),extension=c(0.02,0)))

grid.rect()

grid.points(log10(bacnlsALL$p), bacnlsALL$freq,pch=20,gp=gpar(col=inter.col,cex=0.7))

grid.yaxis()

grid.xaxis()

grid.lines(log10(bacnlsALL$p),bacnlsALL$freq.pred,gp=gpar(col='blue',lwd=2),default='native')

grid.lines(log10(bacnlsALL$p),bacnlsALL$Lower ,gp=gpar(col='blue',lwd=2,lty=2),default='native')

grid.lines(log10(bacnlsALL$p),bacnlsALL$Upper,gp=gpar(col='blue',lwd=2,lty=2),default='native')

grid.text(y=unit(0,'npc')-unit(2.5,'lines'),label='Mean Relative Abundance (log10)', gp=gpar(fontface=2))

grid.text(x=unit(0,'npc')-unit(3,'lines'),label='Frequency of Occurance',gp=gpar(fontface=2),rot=90)

draw.text <- function(just, i, j) {

grid.text(paste("Rsqr=",round(Rsqr,3),"\n","Nm=",round(coef(m.fit)*N)), x=x[j], y=y[i], just=just)

}

x <- unit(1:4/5, "npc")

y <- unit(1:4/5, "npc")

draw.text(c("centre", "bottom"), 4, 1)

#**********************************************************************************************************

######################### Figure6 analysis ###################################

##########################################################################

#########From_Dr.Ning##############

# version 2020.8.23

rm(list=ls())

t0=Sys.time() # to calculate time cost

# 1 # set folder paths and file names, please change according to the folder paths and file names in your computer.

# the folder saving the input files

wd="/Data"

# the OTU table file (Tab delimited txt file)

com.file="otu_table.txt"

# the phylogenetic tree file

tree.file="tree.nwk"

# the treatment informaiton table

treat.file="group.txt"

save.wd="/Data/TestOutputs20"

if(!dir.exists(save.wd)){dir.create(save.wd)}

# 2 # key parameter setting

prefix="Test" # prefix of the output file names. usually use a project ID.

rand.time=1000 # randomization time, 1000 is usually enough. For example test, you may set as 100 or less to save time.

nworker=4 # nworker is thread number for parallel computing, which depends on the CPU core number of your computer.

memory.G=50 # to set the memory size as you need (but should be less than the available space in your hard disk), so that calculation of large tree will not be limited by physical memory. unit is Gb.

# 3 # load R packages and data

library(iCAMP)

library(ape)

setwd(wd)

comm=t(read.table(com.file, header = TRUE, sep = "\t", row.names = 1,

as.is = TRUE, stringsAsFactors = FALSE, comment.char = "",

check.names = FALSE))

tree=read.tree(file = tree.file)

treat=read.table(treat.file, header = TRUE, sep = "\t", row.names = 1,

as.is = TRUE, stringsAsFactors = FALSE, comment.char = "",

check.names = FALSE)

# 4 # match sample IDs in OTU table and treatment information table

sampid.check=match.name(rn.list=list(comm=comm,treat=treat))

# sampid.check=match.name(rn.list=list(comm=comm,treat=treat)) # if you do not have env.file

# for the example data, the output should be "All match very well".

# for your data files, if you have not matched their IDs, the unmatched samples will be removed.

treat=sampid.check$treat

comm=sampid.check$comm

comm=comm[,colSums(comm)>0,drop=FALSE] # if some unmatched samples were removed, some OTUs may become ghosts, then you may use this line to remove them if necessary.

# 5 # match OTU IDs in OTU table and tree file

spid.check=match.name(cn.list=list(comm=comm),tree.list=list(tree=tree))

# for the example data, the output should be "All match very well".

# for your data files, if you have not matched the IDs before, the unmatched OTUs will be removed.

comm=spid.check$comm

tree=spid.check$tree

# 6 # calculate pairwise phylogenetic distance matrix.

# since microbial community data usually has a large number of species (OTUs or ASVs), we use "big.matrix" in R package "bigmemory" to handle the large phylogenetic distance matrix.

setwd(save.wd)

if(!file.exists("pd.desc"))

{

pd.big=iCAMP::pdist.big(tree = tree, wd=save.wd, nworker = nworker, memory.G = memory.G)

# output files:

# path.rda: a R object to list all the nodes and edge lengthes from root to every tip. saved in R data format. an intermediate output when claculating phylogenetic distance matrix.

# pd.bin: BIN file (backingfile) generated by function big.matrix in R package bigmemory. This is the big matrix storing pairwise phylogenetic distance values. By using this bigmemory format file, we will not need memory but hard disk when calling big matrix for calculation.

# pd.desc: the DESC file (descriptorfile) to hold the backingfile (pd.bin) description.

# pd.taxon.name.csv: comma delimited csv file storing the IDs of tree tips (OTUs), serving as the row/column names of the big phylogenetic distance matrix.

}else{

# if you already calculated the phylogenetic distance matrix in a previous run

pd.big=list()

pd.big$tip.label=read.csv(paste0(save.wd,"/pd.taxon.name.csv"),row.names = 1,stringsAsFactors = FALSE)[,1]

pd.big$pd.wd=save.wd

pd.big$pd.file="pd.desc"

pd.big$pd.name.file="pd.taxon.name.csv"

}

####################

setwd(save.wd)

####################

# 9 # iCAMP analysis

# 9.1 # without omitting small bins.

# commonly use # set sig.index as Confidence instead of SES.RC (betaNRI/NTI + RCbray)

bin.size.limit = 12 # For real data, usually use a proper number according to phylogenetic signal test or try some settings then choose the reasonable stochasticity level. our experience is 12, or 24, or 48. but for this example dataset which is too small, have to use 5.

sig.index="Confidence" # see other options in help document of icamp.big.

icres=iCAMP::icamp.big(comm=comm, pd.desc = pd.big$pd.file, pd.spname=pd.big$tip.label,

pd.wd = pd.big$pd.wd, rand = rand.time, tree=tree,

prefix = prefix, ds = 0.2, pd.cut = NA, sp.check = TRUE,

phylo.rand.scale = "within.bin", taxa.rand.scale = "across.all",

phylo.metric = "bMPD", sig.index=sig.index,

bin.size.limit = bin.size.limit,

nworker = nworker, memory.G = memory.G,

rtree.save = FALSE, detail.save = TRUE,

qp.save = FALSE, detail.null = FALSE,

ignore.zero = TRUE, output.wd = save.wd,

correct.special = TRUE, unit.sum = rowSums(comm),

special.method = "depend",

ses.cut = 1.96, rc.cut = 0.95,

conf.cut=0.975, omit.option = "no",meta.ab = NULL)

# there are quite a few parameters in this function, please check the help document of "icamp.big".

# output files:

# Test.iCAMP.detail.rda: the object "icres" saved in R data format. it is a list object. The first element bNRIiRCa is the result of relative importance of each assembly process in each pairwise comparison (each turnover). The second element "detail" including binning information (named taxabin), phylogenetic and taxonomic metrics results in each bin (named like bNRIi, RCa, etc.), relative abundance of each bin (bin.weight), relative importance of each process in each turnover between communities (processes), input settings (setting), and input community data matrix (comm). See help document of the function icamp.big for more details.

############################

# 9.2 to 9.4 are some optional special settings you may explore.

# 9.2 # explore different ways for null model significance test.

# 9.2.1 # set detail.null=TRUE, output all null values, to facilitate normality test and switch between different options

detail.null=TRUE

bin.size.limit =12

sig.index="SES.RC" # this is traditional way, with assumption that null values of phylogenetic metrics follow normal distribution.

prefixb="TestB"

icres2=iCAMP::icamp.big(comm=comm, pd.desc = pd.big$pd.file,

pd.spname=pd.big$tip.label,

pd.wd = pd.big$pd.wd, rand = rand.time, tree=tree,

prefix = prefixb, ds = 0.2, pd.cut = NA, sp.check = TRUE,

phylo.rand.scale = "within.bin", taxa.rand.scale = "across.all",

phylo.metric = "bMPD", sig.index=sig.index,

bin.size.limit = bin.size.limit,

nworker = nworker, memory.G = memory.G,

rtree.save = FALSE, detail.save = TRUE,

qp.save = FALSE, detail.null = detail.null,

ignore.zero = TRUE, output.wd = save.wd,

correct.special = TRUE, unit.sum = rowSums(comm),

special.method = "depend",

ses.cut = 1.96, rc.cut = 0.95, conf.cut=0.975,

omit.option = "no",meta.ab = NULL)

# 9.2.2 # normality test

nntest=iCAMP::null.norm(icamp.output=icres2, p.norm.cut=0.05, detail.out=FALSE)

# output shows non-normal distribution ratio in each bin, i.e. the proportion of turnovers which have null values significantly deviated from normal distribution.

# if some ratio values are very high, may need to change to use "Confidence" as sig.index.

head(nntest$summary)

# 9.2.3 # change sig.index to "Confidence".

icres3=iCAMP::change.sigindex(icamp.output = icres2, sig.index = "Confidence",

detail.save = TRUE, detail.null = FALSE,

conf.cut = 0.975)

head(icres3$CbMPDiCBraya)

# 9.2.4 # change sig.index to "RC" for both phylogenetic and taxonomic metrics.

icres4=iCAMP::change.sigindex(icamp.output = icres2, sig.index = "RC",

detail.save = TRUE, detail.null = FALSE, rc.cut = 0.95)

head(icres4$RCbMPDiRCbraya)

# 9.2.5 # the function can also change the significance threshold.

icres5=iCAMP::change.sigindex(icamp.output = icres2, sig.index = "SES.RC",

detail.save = TRUE, detail.null = FALSE,

ses.cut = 1.64, rc.cut = 0.9)

head(icres5$bNRIiRCbraya)

# 10 # iCAMP bin level statistics

icbin=icamp.bins(icamp.detail = icres$detail,treat = treat,

clas=NULL,silent=FALSE, boot = TRUE,

rand.time = rand.time,between.group = TRUE)

save(icbin,file = paste0(prefix,".iCAMP.Summary.rda")) # just to archive the result. rda file is automatically compressed, and easy to load into R.

write.csv(icbin$Pt,file = paste0(prefix,".ProcessImportance_EachGroup.csv"),

row.names = FALSE)

write.csv(icbin$Ptk,file = paste0(prefix,

".ProcessImportance_EachBin_EachGroup.csv"),

row.names = FALSE)

write.csv(icbin$Ptuv,file = paste0(prefix,".ProcessImportance_EachTurnover.csv"),

row.names = FALSE)

write.csv(icbin$BPtk,file = paste0(prefix,".BinContributeToProcess_EachGroup.csv"),

row.names = FALSE)

# output files:

# Test.iCAMP.Summary.rda: the object "icbin" saved in R data format. see help document of the function icamp.bins for description of each element in the object.

# Test.ProcessImportance_EachGroup.csv: Relative importance of each process in governing the turnovers in a group of samples.

# Test.ProcessImportance_EachBin_EachGroup.csv: Relative importance of each process in governing the turnovers of each bin among a group of samples.

# Test.ProcessImportance_EachTurnover.csv: Relative importance of each process in governing the turnovers between each pair of communities (samples).

# Test.BinContributeToProcess_EachGroup.csv: Bin contribution to each process, measuring the contribution of each bin to the relative importance of each process in the assembly of a group of communities.
